# Response of Arabidopsis primary metabolism and circadian clock to low night temperature in a natural light environment

**DOI:** 10.1093/jxb/ery276

**Published:** 2018-07-25

**Authors:** Maria Grazia Annunziata, Federico Apelt, Petronia Carillo, Ursula Krause, Regina Feil, Karin Koehl, John E Lunn, Mark Stitt

**Affiliations:** 1 Max Planck Institute of Molecular Plant Physiology, Am Mühlenberg, Potsdam-Golm, Germany; 2 University of Campania ‘Luigi Vanvitelli’, Via Vivaldi, Caserta, Italy

**Keywords:** *Arabidopsis thaliana*, core circadian clock genes, fluctuating light, fluctuating temperature, natural environment, starch, sucrose, trehalose 6-phosphate

## Abstract

Plants are exposed to varying irradiance and temperature within a day and from day to day. We previously investigated metabolism in a temperature-controlled greenhouse at the spring equinox on both a cloudy and a sunny day [daily light integral (DLI) of 7 mol m^−2^ d^−1^ and 12 mol m^−2^ d^−1^]. Diel metabolite profiles were largely captured in sinusoidal simulations at similar DLIs in controlled-environment chambers, except that amino acids were lower in natural light regimes. We now extend the DLI12 study by investigating metabolism in a natural light regime with variable temperature including cool nights. Starch was not completely turned over, anthocyanins and proline accumulated, and protein content rose. Instead of decreasing, amino acid content rose. Connectivity in central metabolism, which decreased in variable light, was not further weakened by variable temperature. We propose that diel metabolism operates better when light and temperature are co-varying. We also compared transcript abundance of 10 circadian clock genes in this temperature-variable regime with the temperature-controlled natural and sinusoidal light regimes. Despite temperature compensation, peak timing and abundance for dawn- and day-phased genes and *GIGANTEA* were slightly modified in the variable temperature treatment. This may delay dawn clock activity until the temperature rises enough to support rapid metabolism and photosynthesis.

## Teaser

Central metabolism and the circadian clock are impacted by co-variation in light and temperature in Arabidopsis plants grown in a natural light environment.

## Introduction

In nature, plants live in a fluctuating environment where day is followed by night, and irradiance varies from day to day. Irradiance increases gradually in the morning to reach high intensities during the day, and decreases gradually in the evening, with superimposed and irregular fluctuations on cloudy days. In the daytime, light drives photosynthesis and growth. At night, metabolism, maintenance, and growth depend on reserves that are built up in the light (Smith and Stitt, 2007). In many species, including Arabidopsis (*Arabidopsis thaliana*), the major carbon (C) reserve is starch (Smith and Stitt, 2007). In C-limiting conditions, starch is mobilized at a constant rate at night and is almost exhausted at dawn (Smith and Stitt, 2007; Stitt and Zeeman, 2012). Furthermore, amino acids are accumulated in the daytime to support protein synthesis at night (Pal *et al.*, 2013; Sulpice *et al.*, 2014). Photoperiod duration and irradiance determine the amount of reserves built up, which in turn determines the rate of growth at night (Sulpice *et al.*, 2014; Mengin *et al.*, 2017).

In the field, changes in irradiance are usually accompanied by changes in temperature. These modify growth responses; for example, low temperature decreases consumption of reserves for maintenance and growth at night (Larcher, 1995; Gillooly *et al.*, 2001; Atkin and Tjoelker, 2003; Pyl *et al.*, 2012; Pilkington *et al.*, 2015). Temperature fluctuates on different time scales: within minutes due to changes in irradiance, hours as a result of the diel light/dark cycle, days due to changing weather, and weeks to months due to season (Larcher, 1995). Temperature changes are typically slightly delayed compared with changes in irradiance (Matsuzaki *et al.*, 2015).

Plants, like other life forms, have evolved an internal timekeeper to predict and respond to rhythmic changes in the environment (Locke *et al.*, 2005; McClung, 2006; Harmer, 2009; Song *et al.*, 2010). The Arabidopsis circadian clock is a highly interconnected network with successive expression of dawn (*LATE ELONGATED HYPOCOTYL*, *LHY*; *CIRCADIAN CLOCK ASSOCIATED1*, *CCA1*), day (*PSEUDO-RESPONSE REGULATOR9*, *PRR9*; *PRR7*), dusk (*PRR5*; *TIMING OF CAB EXPRESSION1*, *TOC1*; *GIGANTEA*, *GI*), and evening (*LUX ARRHYTHMO*, *LUX*; *EARLY FLOWERING4*, *ELF4*; *ELF3*) components (Pokhilko *et al.*, 2012; Fogelmark and Troein, 2014). It operates with an endogenous periodicity of ~24 h and is entrained to dawn by light signalling (Edwards *et al.*, 2010; Kinmonth-Schultz *et al.*, 2013; Seo and Mas, 2014). It is largely temperature compensated, whilst having the ability to be entrained by temperature changes (Gould *et al.*, 2006; McClung and Davis, 2010; Salomé *et al.*, 2010; Mizuno *et al.*, 2014; Kidd *et al.*, 2015). These features align the internal clock cycle to the external light–dark cycle across a wide range of environmental conditions.

The circadian clock plays a key role in the regulation of diel starch turnover, especially the pacing of starch mobilization to dawn (Lu *et al.*, 2005; Graf *et al.*, 2010; Graf and Smith, 2011; Greenham and McClung, 2015). Scialdone *et al.* (2013) proposed that clock regulation of starch degradation occurs by arithmetic division; the clock provides information about time to dawn that is integrated with a measure of starch content to set a degradation rate such that starch is almost exhausted by the next dawn. Other models involving metabolic feedback into the clock have also been proposed (Dodd *et al.*, 2014; Webb and Satake, 2015; Seki *et al.*, 2017). It is plausible that multiple mechanisms regulate diel starch turnover, but our mechanistic understanding is incomplete. The circadian clock also contributes to diel regulation of organic acid and amino acid metabolism (Gutierrez *et al.*, 2008; Fukushima *et al.*, 2009; Espinoza *et al.*, 2010), but our mechanistic understanding is even more rudimentary than for starch.

Most plant metabolic studies are performed in controlled environments with regular recurring diel changes. It is important to learn how plants orchestrate metabolism in a naturally varying environment (Nagano *et al.*, 2012; Matsuzaki *et al.*, 2015). Recently, we compared diel metabolism in Arabidopsis grown in a naturally illuminated greenhouse around the spring equinox in Golm and in controlled-environment chambers in a 12 h photoperiod with square-wave or sinusoidal light profiles (Annunziata *et al.*, 2017*a*). We found that organic acid and amino acid metabolism are less robust than starch turnover to a fluctuating light regime. We now extend this study by investigating diel metabolism under natural changes in light and varying temperature, and diel changes of 10 clock transcripts in temperature-controlled sinusoidal and natural light regimes, and in the natural light regime with varying temperature.

## Materials and methods

### Plant material and growth conditions

Wild-type *Arabidopsis thaliana* Columbia-0 (MPI-MP in-house collection) was germinated on 1:1 soil:vermiculite mix in a 16 h photoperiod (250 µmol m^−2^ s^−1^, 20 °C/6 °C light/dark, 75% humidity), transferred 7 days after sowing (DAS) to an 8 h photoperiod (100 µmol m^−2^ s^−1^, 20 °C/60% humidity in light, 16 °C/75% humidity at night), transplanted at 14 DAS to 10 cm diameter pots (five plants per pot; soil as for germination), and placed in one of the following regimes. (i) Controlled light and temperature (LT): controlled-environment chamber (Percival E-36 L; CLF Plant Climatics, Wertingen, Germany; fluorescent light, for spectra see Annunziata *et al.*, 2017*a*), 12 h photoperiod with a sinusoidal regime peaking at 465 µmol m^−2^ s^−1^ (equivalent to the average between ZT4 and ZT8 in L^VAR^T), daily light integral (DLI) 12 mol m^−2^ d^−1^, temperature 21–22 °C in light, 20 °C at night, 65–75% relative humidity. (ii) Naturally variable light and controlled temperature (L^VAR^T): naturally illuminated glasshouse (location 52°24'55.2''N, 12°58'5.5''E) near the 2015 spring equinox, average temperature 22 °C in the day, 20 °C at night, 24 h temperature range 3–4K (see Supplementary Table S1 at *JXB* online). (iii) Naturally variable light and variable temperature (L^VAR^T^VAR^): naturally illuminated greenhouse (located 200 m from the glasshouse) with a polyethylene shell [Lumisol clear AF (Folitec), Lumitherm UV5 (Folitec) on vents], no temperature control except frost protection heating set to ~10 °C, near the 2015 spring equinox; in the 2 weeks preceding harvest, minimum temperatures 10–13 °C, maximum temperatures 16–30 °C, diel temperature range 4–19K. Irradiances in the immediately preceding 24 h cycle and harvest cycle are shown in Supplementary Fig. S1. A summary of the climate data for the preceding 2 weeks in L^VAR^T and L^VAR^T^VAR^ is presented in Supplementary Table S1. L^VAR^T and L^VAR^T^VAR^ were performed in parallel. LT was performed later, with a sinusoidal irradiance profile designed to simulate conditions in the glasshouse on harvest day. Due to cool night temperature, thermal sum [cumulative sum of (temp_min + temp_max)/2] over 14 d preceding harvest was lower in L^VAR^T^VAR^ (233 °C) than in L^VAR^T (303 °C) or LT (294 °C; Supplementary Table S1). On harvest day, the DLI (total irradiance per day) was 12.8, 11.7, and 14 mol m^−2^ d^−1^ in LT, L^VAR^T, and L^VAR^T^VAR^, respectively (slightly lower in L^VAR^T due to shading by internal structures). Light intensity and quality were measured using a Spectis 1.0 Touch (GL Optic Lichmesstechnik, Weilheim, Germany).

At 28 DAS, plants were harvested at 2 h intervals over a 24 h cycle, with more frequent sampling (0.5–1 h) around dawn and dusk. At each time, four replicates (five rosettes per sample) were frozen in liquid nitrogen. Samples were collected in <5 min by different harvesters at the different locations. Samples were powdered and subaliquoted at −70 °C in an automated cryogenic robot (Stitt *et al.*, 2007) and stored at −80 °C.

### Biomass

FW and DW (dry for 48 h at 70 °C) were determined on an analytical balance (accuracy 0.001 g). Relative water content was calculated as in Annunziata *et al.* (2017*b*).

### Metabolites

Metabolites were extracted and assayed as in Annunziata *et al.* (2017*a*), and anthocyanins as in Nakata *et al.* (2013).

### qRT-PCR

Total RNA was isolated and cDNA prepared and quality tested as in Flis et al. (2015, 2016). qRT-PCR was performed essentially as in Flis *et al.* (2015, 2016) using reference genes listed in Supplementary Table S2 for normalization.

### Statistical analyses

Statistical analyses were performed with ANOVA and pairwise Student’s *t*-test functions of SigmaPlot 12.5 (Systat Software GmbH, Erkrath, Germany; www.systat.de). Principal component analysis (PCA) was performed using XLStat-Base (Addinsoft, New York, USA; www.xlstat.com) software. Quality control and descriptive statistics on meteorological data were calculated in SAS 9.4 (SAS Institute). The effects of experimental conditions (LT, L^VAR^T, and L^VAR^T^VAR^) and sampling time [Zeitgeber time (ZT)] on abundance of circadian clock transcripts were determined by two-way ANOVA to detect genes whose expression pattern was altered by experimental conditions or an interaction of experimental condition and time, followed by a post-hoc Ryan–Einot–Gabriel–Welch (REGW) test, SAS 9.4 for significant differences in expression time series between different conditions and for the timing of transcript peak time (SAS Institute; see Supplementary Table S7 for details).

## Results

### Experimental design

Arabidopsis Col-0 plants were grown close to the spring equinox in 2015 in a glasshouse with natural light and controlled temperature (22 °C light period, 20 °C night, L^VAR^T) or a polythene greenhouse where they were exposed to natural light and ambient temperature, except that supplementary heating was to prevent the temperature falling below 10 °C (L^VAR^T^VAR^). During the 14 d preceding harvest, the main difference between L^VAR^T^VAR^ and L^VAR^T was a more variable and larger temperature range (4–20 °C and 3–4 °C, respectively) and lower night temperature (10–13 °C and 20 ± 1 °C, respectively). L^VAR^T received slightly less cumulative light than L^VAR^T^VAR^ because the glasshouse cabin was sometimes shaded to prevent the temperature rising. This did not occur on the harvest day. A third set of plants was grown later in a controlled-environment chamber with white fluorescent tubes operated in a sinusoidal light regime and controlled temperature (LT) designed to simulate the daily light integral (DLI, total light received) on the harvest day in the temperature-controlled glasshouse.

Supplementary Fig. S2 shows images of representative plants on harvest day (28 DAS), and rosette FW and DW. Supplementary Table S3 provides data for rosette FW and DW and water content in L^VAR^T and L^VAR^T^VAR^ plants, measured at 3 h intervals on harvest day. LT and L^VAR^T^VAR^ plants had a similar developmental stage (10–12 leaves, non-flowering), rosette diameter, FW, and DW. L^VAR^T plants were smaller, probably due to them receiving less light earlier in their life history (see above). Water content at ZT12 was slightly but non-significantly higher in L^VAR^T (90.6 ± 0.78%) than in L^VAR^T^VAR^ (88.7 ± 1.16%) or LT (87.4 ± 2.89%).

Plants were harvested over a 24 h light–dark cycle to analyse metabolites (data in Supplementary Table S4) and circadian clock transcript abundance (data in Supplementary Table S5). Time-series were aligned to dawn (ZT0), defined in the sinusoidal regime as the first light provided (intensity 11.75 µmol m^−2^ s^−1^) and in the natural light regimes as sunrise (Supplementary Fig. S1).

### Principal component analysis

**Fig. 1.**
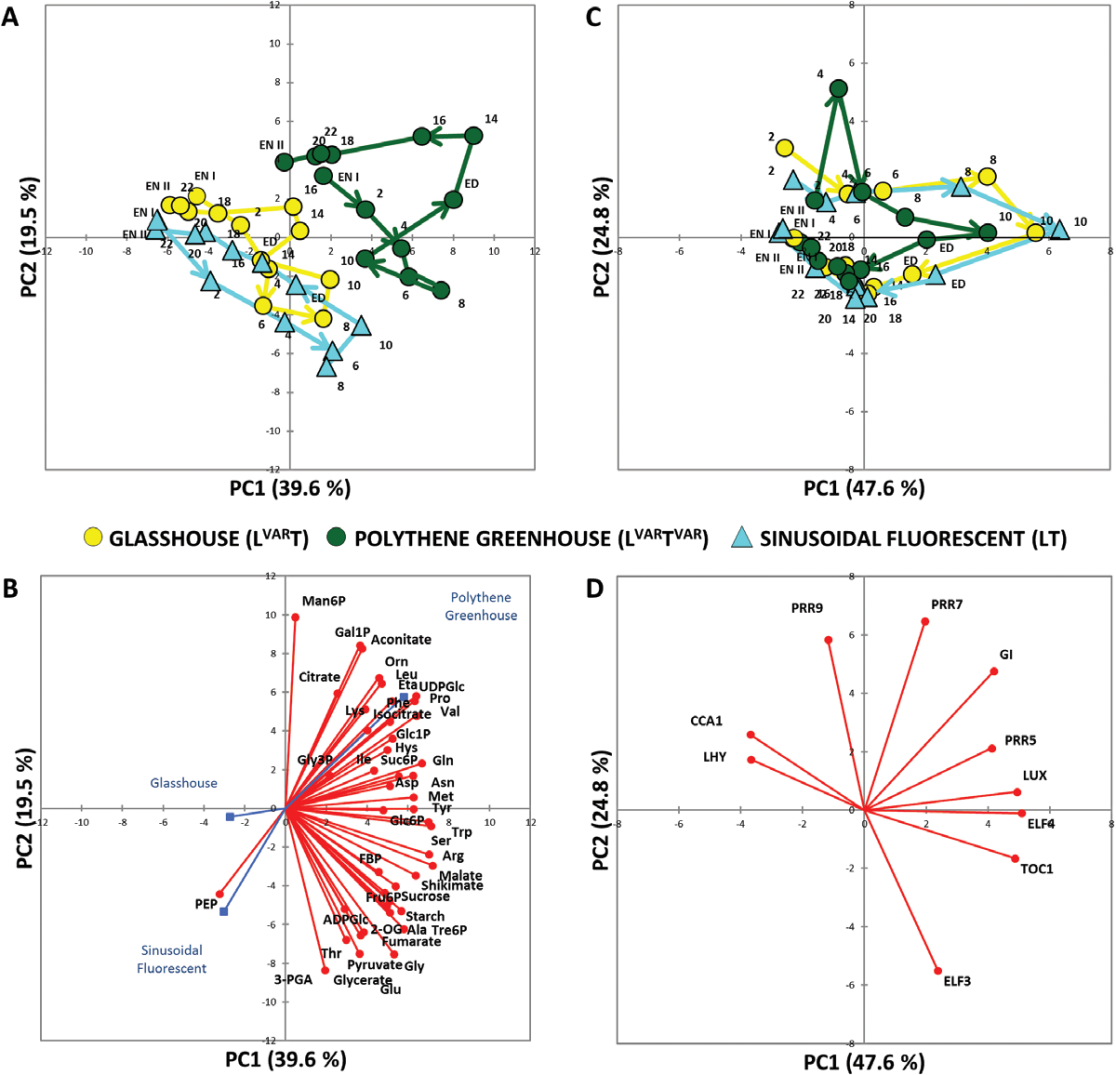
Principal component analysis (PCA) of metabolite and core circadian clock gene data from Arabidopsis plants. PCA of metabolite data (A) and of core clock genes (C) from plants grown around the vernal equinox in 2015 in a naturally illuminated and temperature-controlled glasshouse (L^VAR^T, yellow circles) or a naturally illuminated polythene greenhouse with a less controlled temperature (L^VAR^T^VAR^, green circles). Plants were also grown in a controlled-environment chamber with a 12 h photoperiod and daily light integral (DLI) of 12 mol m^−2^ d^−1^. The artificial illumination was provided by white fluorescent tubes with a sinusoidal (LT, cyan triangles) light profile during the day. Numbers indicate the time of harvest in hours after dawn (Zeitgeber time, ZT); ED, end of day (ZT12); EN I, end of preceding night (ZT0); EN II, end of night (ZT24); and the diurnal trajectories are indicated by arrows. The percentages of total variance represented by principal component 1 (PC1) and principal component 2 (PC2) are shown in parentheses. (B and D) The loadings of individual metabolites or genes in PC1 and PC2. FBP, fructose 1,6-bisphosphate; Eta, ethanolamine; Orn, ornithine.

To provide a first overview, we performed PCA. In the analysis of the metabolite data set (Fig. 1A), PC1 and PC2 accounted for 40% and 20% of total variation, respectively. LT (cyan triangles) and L^VAR^T (yellow circles) plants showed overlapping diel trajectories. L^VAR^T^VAR^ showed a strongly displaced trajectory, and also showed larger differences between daytime and night-time samples than the other regimes. Figure 1B shows the underlying variables (metabolite abundances). Many metabolites drove the diel changes including starch, sucrose, trehalose 6-phosphate (Tre6P; a sucrose signalling metabolite), pyruvate, malate, fumarate, shikimate (precursor of aromatic amino acids), 2-oxoglutarate (2OG), and several amino acids (including Glu, Gly, Ser, Asp, Ala, and Thr). Displacement of L^VAR^T^VAR^ from the other regimes was driven by citrate, isocitrate, aconitate, Gln, Phe, Leu, Ile, Val, Pro, UDP-glucose (UDPGlc), Glc1P (glucose 1-phosphate), mannose 6-phosphate (Man6P), and galactose 1-phosphate (Gal1P) (see below for further data and discussion).

In the analysis of clock transcript abundance (Fig. 1C), PC1 and PC2 accounted for 48% and 25% of the variation, respectively. The trajectories of the three regimes overlapped. L^VAR^T^VAR^ nevertheless showed a divergence at ZT4 and a partly opposing divergence at ZT6–10. Trajectories were rather similar at night, when temperature was cooler in L^VAR^T^VAR^. Figure 1D shows the underlying variables (transcript abundances). Their orientation roughly follows the diel timing of their expression peaks (Pokhilko *et al.*, 2012; Flis *et al.*, 2015; and see below). The divergence of L^VAR^T^VAR^ at ZT4 is associated with the vectors for *PRR9* and *PRR7*. PCA on the L^VAR^T^VAR^ data set alone (Supplementary Fig. S3) confirmed that ZT4 was strongly separated from other time points.

Overall, the sinusoidal simulation largely recapitulates the diel responses of metabolism and the clock in a natural light regime provided temperature is controlled. Varying temperature has a larger impact than varying light on both metabolism and the transcriptional clock, with the former being particularly affected.

### Heat map to display differences in metabolite levels between treatments

**Fig. 2.**
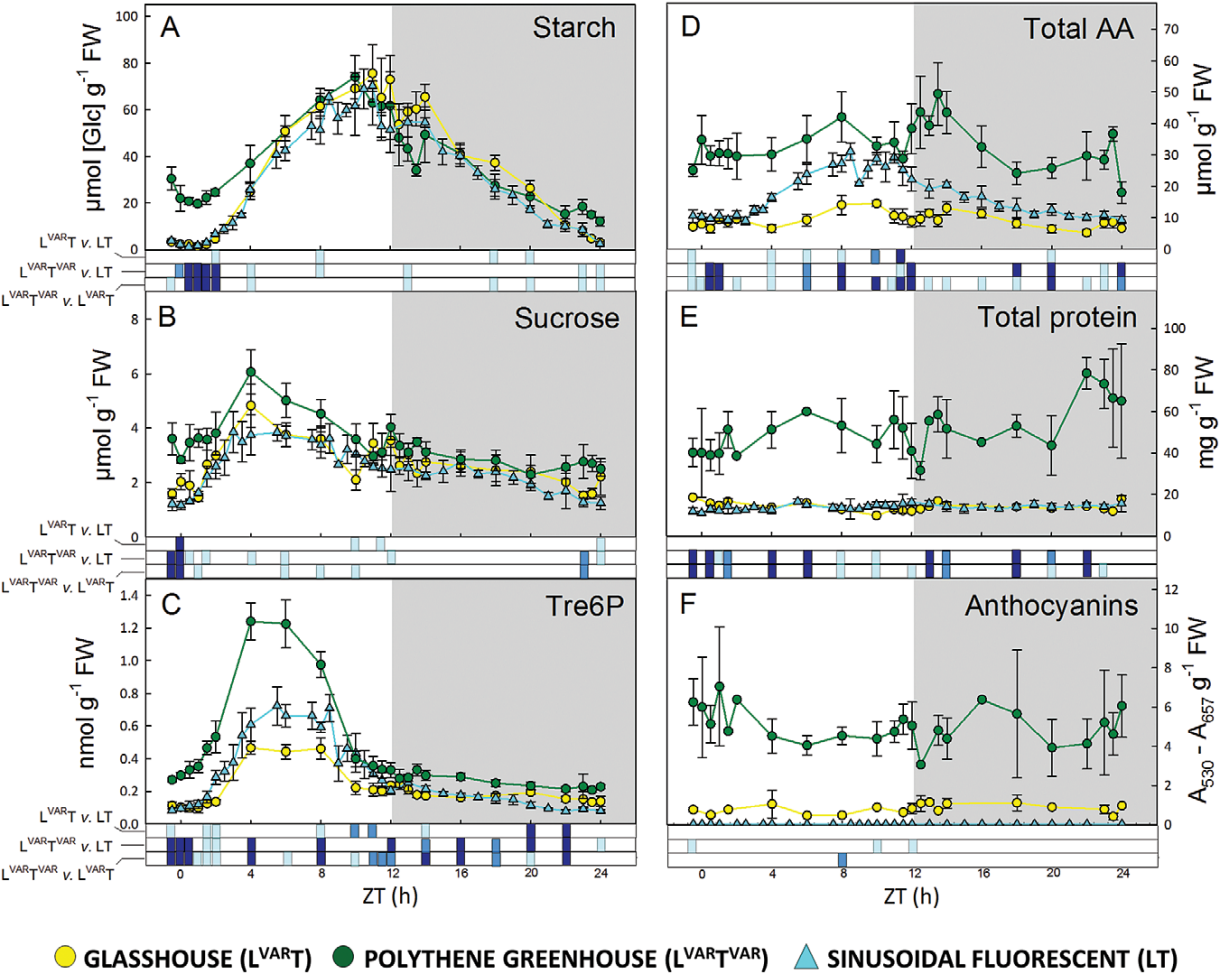
Diurnal profiles of metabolites in Arabidopsis plants growing in natural or fluorescent light with a 12 h photoperiod at DLI 12. *Arabidopsis thaliana* Col-0 plants were grown in a naturally illuminated and temperature-controlled glasshouse (L^VAR^T, yellow circles) or a naturally illuminated polythene greenhouse with a less controlled temperature (L^VAR^T^VAR^, green circles) around the vernal equinox in 2015. Plants were also grown in a controlled-environment chamber with a 12 h photoperiod and DLI of 12 mol m^−2^ d^−1^. Artificial illumination was provided by white fluorescent tubes with a sinusoidal light profile (LT, cyan triangles). Rosettes were harvested from 4-week-old plants throughout a 24 h diurnal cycle for analysis of: (A) starch, (B) sucrose, (C) Tre6P, (D) total amino acids, (E) total protein, and (F) anthocyanins. Data are the mean ±SD (*n*=4). At each time point, significant differences between each pair of growth regimes are indicated by different colours: *P*<0.05=light blue, *P*<0.01=blue, *P*<0.001=dark blue. ZT, Zeitgeber time (hours after dawn).

**Fig. 3.**
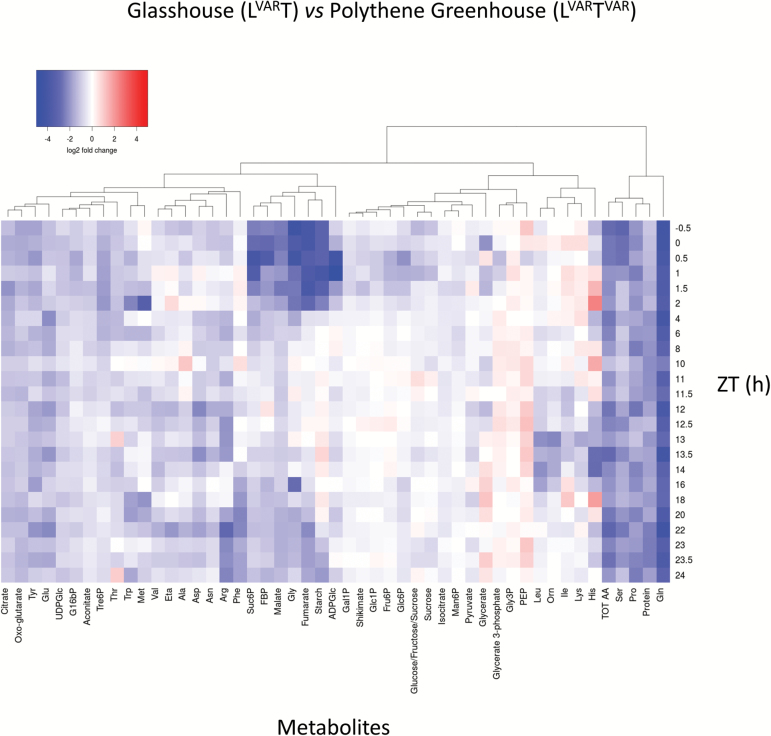
Log_2_ fold changes of the relative metabolic content are presented as a heat map of glasshouse (L^VAR^T) versus polythene greenhouse (L^VAR^T^VAR^). Data are clustered based on hierarchical agglomerative clustering with complete linkage. Colour key: low values, blue; high values, red. FBP, fructose 1,6-bisphosphate; Eta, ethanolamine; Orn, ornithine.

Many metabolites with a high weighting in PC1 were low at dawn and high towards the end of the light period; examples included starch, sucrose, Tre6P (Fig. 2A-C), malate, fumarate, 2OG (Supplementary Fig. S4B), Glu, Gly, Ser, Asp, Ala (Supplementary Fig. S4C), and Thr (Supplementary Fig. S4E). A statistical analysis is provided in Supplementary Table S6. To disentangle regime-dependent changes from diel changes, we calculated the change in L^VAR^T^VAR^ relative to L^VAR^T or LT for each metabolite and time point, transformed the ratios to a log_2_ scale, clustered them, and displayed the matrix as a heat map (Fig. 3; Supplementary Fig. S5A; note that time points are ordered from top to bottom starting 0.5 h before dawn). A similar display was made for L^VAR^T relative to LT (Supplementary Fig. S5B).

As in the PCA, the most pronounced differences were found for the L^VAR^T versus L^VAR^T^VAR^ (Fig. 3) and LT versus L^VAR^T^VAR^ (Supplementary Fig. S5A) comparisons. Many traits were higher over the entire diel cycle in L^VAR^T^VAR^, including protein, total amino acids, Gln, Ser, Pro, and, to a lesser extent, Tre6P, UDPGlc, citrate, 2OG, aconitate, Glu, Asp, Asn, Arg, and Thr. There were also time-of-cycle-dependent differences between L^VAR^T^VAR^ and the other treatments; for example, higher starch, malate, fumarate, Gly, sucrose 6-phosphate (Suc6P), and fructose 1,6-bisphosphate (Fru1,6BP) between ZT0.5 and ZT2 and, though less marked, ZT20 and ZT24 (Fig. 3; Supplementary Fig. S5A).

Fewer differences were observed in the L^VAR^T versus LT comparison (Supplementary Fig. S5B). Gln was lower throughout the diel cycle in L^VAR^T. Asp was only slightly higher, and other amino acids including Ser, Glu, Asn, Ala, and Arg were similar in these two conditions at ZT0–ZT2, but were lower in L^VAR^T than in LT later in the diel cycle; this is because amino acids increase less strongly in the light period in L^VAR^T than in LT (Supplementary Fig. S4C–E). Leu, Lys, and Orn were higher in L^VAR^T.

### Correlation analysis to compare diel changes in different regimes

**Fig. 4.**
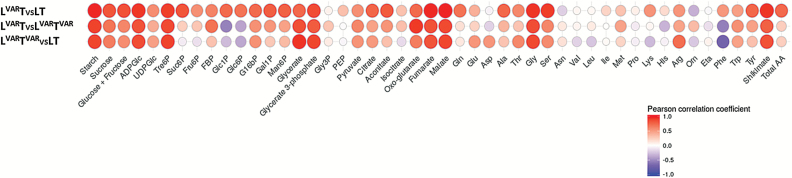
Correlation analysis of pair-wise comparison of metabolite time series. A Pearson correlation coefficient was calculated for each pair of conditions based on the positive or negative correlation between the metabolite profiles over the entire light–dark cycle. The Pearson correlation coefficient ranges from –1 (blue) to 1 (red), with –1 indicating higher negative correlation and 1 indicating higher positive correlation between the two compared data sets. FBP, fructose 1,6-bisphosphate; Eta, ethanolamine; Orn, ornithine.

To compare diel changes across regimes, we calculated, for each metabolite time-series, the Pearson correlation coefficient between each pair of regimes (Fig. 4). When LT and L^VAR^T were compared, many metabolites showed correlated diel changes; out of 48 metabolites, 38 had a positive correlation with *R*>0.3, and four (Asn, Orn, Asp, and ethanolamine) showed a negative correlation. Starch, fumarate, and malate showed the highest positive correlations (≥0.98). When L^VAR^T^VAR^ was compared with LT or L^VAR^T, many metabolites showed differing diel changes. This was especially so for N-containing metabolites; out of 21 amino acids, in the L^VAR^T^VAR^ versus L^VAR^T comparison only nine had a positive correlation >0.3 and four were negatively correlated, and in the L^VAR^T^VAR^ versus LT comparison only six amino acids had a positive correlation >0.30 and seven were negatively correlated (see the Discussion).

### Correlation analysis to compare metabolic connectivity in different growth regimes

**Fig. 5.**
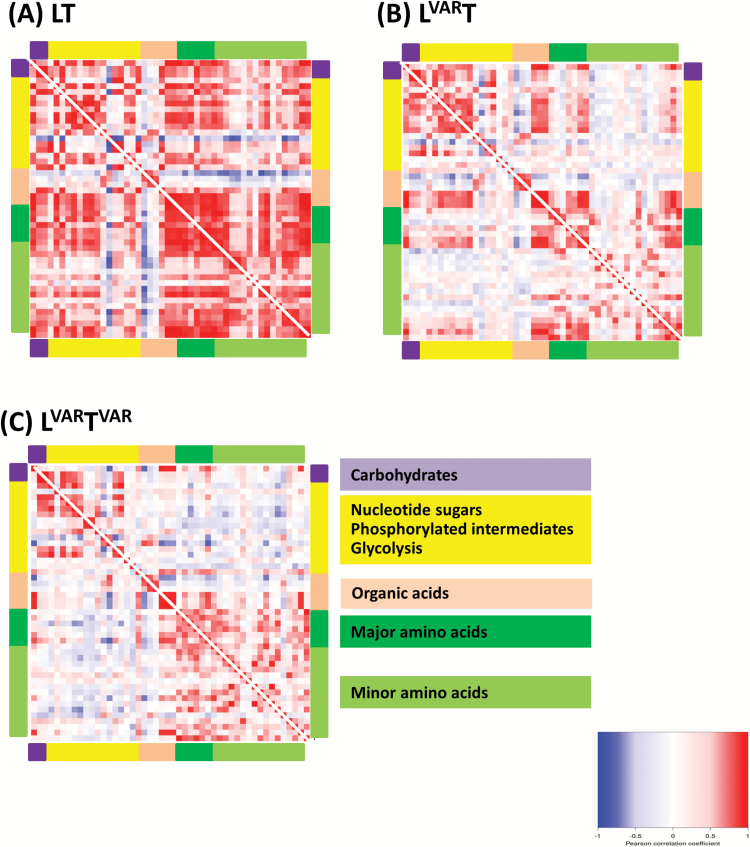
Connectivity in metabolism in different growth regimes. In a given growth regime, each metabolite diel time series was regressed against every other metabolite diel time series. The results are presented as a heat map with Pearson correlation coefficients (*R*) indicated by the shading: red, positive correlation; blue, negative correlation. The colour scale is logarithmic and was chosen such that the majority of non-significant correlations (*P*<0.05) were not assigned any colour. Metabolites are grouped by pathway indicated by the coloured bars at the side of the heat maps. Expanded displays with information on individual metabolites are provided in Supplementary Fig. S6.

We next asked whether metabolites showed co-ordinated diel changes in a given regime, and if the extent of co-ordination varied between growth regimes. For this purpose, we performed a metabolite–metabolite correlation analysis within each regime. The results were visualized as a heat map, in which each square area represents the Pearson correlation coefficient (*R*) between the diel changes of a pair of metabolites; Fig. 5 shows a compact overview and Supplementary Fig. S6 provides a larger display with annotation of individual metabolites.

The strongest connectivity was found in LT (Fig. 5; Supplementary Fig. S6A) where almost all metabolites correlated positively with each other. Exceptions included Lys, Man6P, aconitate, isocitrate, and citrate, which often showed negative correlations to all the other metabolites. L^VAR^T showed weaker connectivity (Fig. 5; Supplementary Fig. S6B); strong positive correlations were largely restricted to carbohydrates and phosphorylated intermediates, some tricarboxylic acid (TCA) cycle intermediates (2OG, malate, and fumarate), some amino acids (Ala, Gly, Ser, Trp, and Tyr), and shikimate. L^VAR^T^VAR^ showed weaker metabolite–metabolite correlations than LT but, despite the more strongly fluctuating environment, connectivity was not weaker than in L^VAR^T. There were still positive correlations among carbohydrates and phosphorylated intermediates, and among amino acids, with the notable exceptions of Pro and Phe (Supplementary Fig. S6C).

### Impact of variable temperature on diel changes of starch and selected metabolites

We next inspected responses of individual metabolites. Diel starch turnover was similar in L^VAR^T and LT (Fig. 2A). After a lag of ~2 h, starch accumulated linearly until about ZT8, plateaued or started to decline in the last 2 h of the light period, continued to decline in the night, and was almost exhausted by the next dawn. Fluctuations around dusk probably reflect biological noise. Diel starch turnover was very different in L^VAR^T^VAR^. Compared with temperature-controlled regimes, starch was 10-fold higher at dawn (30–20 μmol [Glc] g^−1^ FW) in L^VAR^T^VAR^. Starch continued to decline until ZT2, started to accumulate after ZT4 but more slowly than in LT and L^VAR^T, plateaued towards the end of the light period at similar levels to the other regimes, and was degraded more slowly at night. The starch excess phenotype in L^VAR^T^VAR^ was stronger at the first dawn than at the end of the harvested cycle (on average, ~2-fold higher, *P*=0.035) (Fig. 2A); this may be due to higher and more stable irradiance on the preceding day, Supplementary Fig. S1). Rates of accumulation and mobilization were estimated by fitting a linear function to the daytime and night-time points. Starch accumulation was faster in L^VAR^T and LT (6.7 µmol [Glc] g^−1^ FW h^−1^ and 6.1 µmol [Glc] g^−1^ FW h^−1^, respectively, over the entire light period; 9.8 µmol [Glc] g^−1^ FW h^−1^ and 8.5 µmol [Glc] g^−1^ FW h^−1^ between ZT2 and ZT8) than L^VAR^T^VAR^ (4.2 µmol [Glc] g^−1^ FW h^−1^ over the entire light period and 6.6 µmol [Glc] g^−1^ FW h^−1^ between ZT2 and ZT8). Starch mobilization was similar in L^VAR^T and LT (–5.2 µmol [Glc] g^−1^ FW h^−1^ and –4.9 µmol [Glc] g^−1^ FW h^−1^), and slower in L^VAR^T^VAR^ (–2.9 µmol [Glc] g^−1^ FW h^−1^).

Sucrose levels in LT and L^VAR^T tracked irradiance, with a rise until about ZT8 followed by a decline (Fig. 2B). Sucrose was slightly, but consistently, higher in L^VAR^T^VAR^ than in the temperature-controlled treatments in the last part of the night, much higher in the first hours after dawn, and slightly higher later in the light period, with the difference disappearing at dusk. L^VAR^T^VAR^ plants also had higher levels of many phosphorylated intermediates (Supplementary Fig. S4A) during the first 2 h of the day, and in many cases until ZT8; for example, Fru1,6BP, glucose 1,6-bisphosphate (Glc1,6BP), and Suc6P were significantly higher than in the temperature-controlled regimes (Supplementary Table S6). UDPGlc was 2- to 3-fold higher than the other treatments (data not plotted; see Supplementary Table S4). TCA cycle intermediates were also significantly higher in L^VAR^T^VAR^ than in the temperature-controlled regimes (Supplementary Fig. S4B; Supplementary Tables S4, S6) with the exception of isocitrate. Citrate and aconitate were higher in L^VAR^T^VAR^ for most of the diel cycle, except around dusk and early in the night. A similar pattern was seen for 2OG, malate, and fumarate compared with L^VAR^T, but not compared with LT, which had higher levels of these organic acids in the middle of the diel cycle.

L^VAR^T plants contained lower total amino acids than LT plants (Fig. 2D), due to low levels of Gln and most other individual amino acids (Supplementary Fig. S4C–E; see also Annunziata *et al.*, 2017*a*). L^VAR^T^VAR^ plants showed large differences in N metabolism compared with the temperature-controlled regimes. Strikingly, the differences were opposite to those in L^VAR^T. Total amino acids were 3–5 and 2–4 times higher in L^VAR^T^VAR^ than in LT or L^VAR^T, respectively (Fig. 2D). Gln was up to 6-fold higher in L^VAR^T^VAR^ than in the other regimes (Supplementary Fig. S4C). The high Gln quantitatively accounts for most of the high total amino acids in L^VAR^T^VAR^. As Glu was not greatly affected, there was a large increase of the Gln:Glu ratio in L^VAR^T^VAR^ (Supplementary Fig. S4C). There was up to 10-fold higher Pro throughout the diel cycle in L^VAR^T^VAR^ (Supplementary Fig. S4E). Other amino acids showed time-of-cycle increases in L^VAR^T^VAR^ compared with the other regimes; Trp was 2-fold higher for much of the light period and in the middle of the night (Supplementary Fig. S4D). Many minor amino acids (Phe, Arg, Lys, His, Orn, Val, Leu, and Ile) were higher at or after dusk (Supplementary Fig. S4D, E), and several major amino acids (Asp, Gly, Ser, and Asn) were higher late in the night and in the first 2 h after dawn (Supplementary Fig. S4C). This presumably reflects decreased amino acid consumption in the cool night (see the Discussion).

### Trehalose 6-phosphate

Tre6P is a signal of sucrose availability (Lunn *et al.*, 2006, 2014) and tracked sucrose (Fig. 2C) as predicted by the sucrose–Tre6P nexus model (Yadav *et al.*, 2014). Both metabolites rose between dawn and the middle of the light period and fell as dusk approached, and both were higher in L^VAR^T^VAR^ than in temperature-controlled treatments. However, there were quantitative differences. Tre6P increased more strongly than sucrose in L^VAR^T^VAR^ in the middle of the light period relative to the levels earlier and later in the day. Tre6P was also higher relative to sucrose in L^VAR^T^VAR^ in the middle of the light period compared with the temperature-controlled regimes at the same time. There was a highly significant positive correlation between Tre6P and sucrose in all three treatments, both in the light and at night (Supplementary Fig. S7). However, in the light, the slope was slightly steeper in L^VAR^T^VAR^ and lower in L^VAR^T than in LT. In the dark the Tre6P:sucrose ratio was shifted upwards in L^VAR^T^VAR^ compared with LT or L^VAR^T. This contrasts with the lower Tre6P:sucrose ratio found in Arabidopsis growing at a constant 8 °C compared with 20 °C (Carillo *et al.*, 2013).

### Protein content and anthocyanins

Low temperatures lead to high protein content (Strand *et al.*, 1999; Stitt and Hurry, 2002; Usadel *et al.*, 2008*a*) and high anthocyanin (Nozzolillo *et al.*, 1990; Christie *et al.*, 1994; Leng *et al.*, 2000). L^VAR^T^VAR^ plants had 4–8 times higher protein content than plants in temperature-controlled regimes (Fig. 2E). The reasons why rosette protein content rose despite the likelihood that protein synthesis is restricted at night by low temperature will be given in the Discussion.

L^VAR^T^VAR^ plants showed localized red-purple coloration, and contained up to 10 or 300 times more anthocyanins than L^VAR^T or LT plants, respectively. Incidentally, the 30-fold higher anthocyanins in L^VAR^T compared with LT (Fig. 2F) were the largest metabolic difference observed between the two temperature-controlled regimes.

### C-starvation marker transcripts

A high Gln/Glu ratio and high Asn level can be indicative of C starvation (Lam *et al.*, 1996). We checked whether C starvation responses were activated in L^VAR^T^VAR^ by analysing transcript abundance for two C starvation-induced (*ATG8e* and *BCAT-2*) and two C starvation-repressed (*CPN60ALPHA1* and *CPN60BETA2*) genes (Bläsing *et al.*, 2005; Osuna *et al.*, 2007). Transcript abundance did not differ between L^VAR^T^VAR^ and the temperature-controlled regimes (Supplementary Fig. S8).

### Core clock transcript abundance in fluctuating environments

**Fig. 6.**
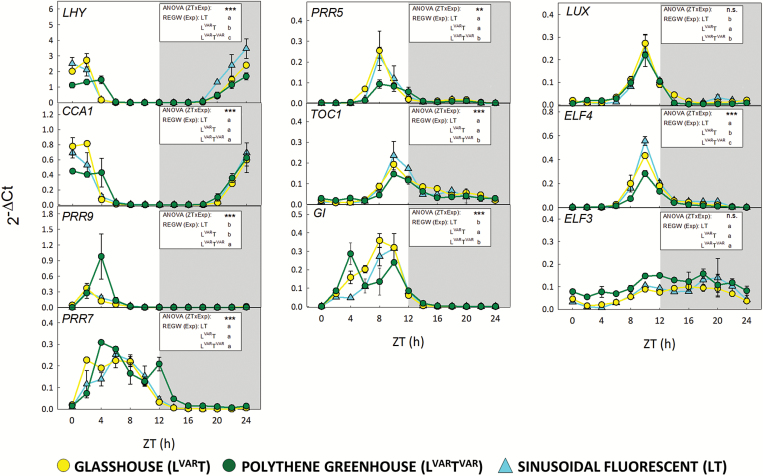
Diurnal gene expression of core circadian clock genes in Arabidopsis plants growing in natural or fluorescent light with a 12 h photoperiod at DLI 12. *Arabidopsis thaliana* Col-0 plants were grown in a naturally illuminated and temperature-controlled glasshouse (L^VAR^T, yellow circles) or a naturally illuminated polythene greenhouse with a less controlled temperature (L^VAR^T^VAR^, green circles) around the vernal equinox in 2015. Plants were also grown in a controlled-environment chamber with a 12 h photoperiod and DLI of 12 mol m^−2^ d^−1^. Artificial illumination was provided by white fluorescent tubes with a sinusoidal light profile (LT, cyan triangles). Rosettes were harvested from 4-week-old plants throughout a 24 h diurnal cycle for expression analysis of core clock genes. The relative expression values were calculated as 2^−ΔCt^ for each sample where ΔCt indicates the difference from the Ct values of the different tested genes and the geometric mean of the Ct values of all reference genes. Data are the mean ±SD (*n*=2) and are shown on a linear scale. Two-way ANOVA (proc GLM, SAS 9.4) was performed on each gene for the factors sampling time (ZT), experimental condition (Exp), and their interaction effect (ZT×Exp). Significances of the interaction effect [ANOVA (ZTxExp)] are shown in each panel indicated by asterisks: **P<0.001, ***P=0.0001, n.s.=not significant. Pairwise comparison for each gene for the means of the factor Exp [REGW (Exp)] is also shown in each panel. Significant differences are indicated by different letters. See Supplementary Table S7 for detailed description of the statistical procedures, and Supplementary Figs S9 and S10 for a display and tests on peak times. ZT, Zeitgeber time (hours after dawn), Exp, experimental treatment. *LHY*, *LATE ELONGATED HYPOCOTYL*; *CCA1*, *CIRCADIAN CLOCK ASSOCIATED1*; *PRR9*, *PRR7*, *PRR5*, *PSEUDO-RESPONSE REGULATOR9*, *7*, and *5*; *TOC1*, *TIMING OF CAB EXPRESSION1*; *GI*, *GIGANTEA*; *LUX*, *LUX ARRHYTHMO*; *ELF4*, *ELF3*, *EARLY FLOWERING4* and *3.*

Ten clock transcripts (*LHY*, *CCA1*, *PRR9*, *PRR7*, *PRR5*, *TOC1*, *GI*, *LUX, ELF4*, and *ELF3*) were measured in samples taken at 2 h intervals in the three growth regimes. Diel changes in abundance are shown in Fig. 6 (linear scale; shows changes in peak values), Supplementary Fig. S9 (log scale; shows differences in troughs), and Supplementary Fig. S10 (radar plots; visualizes peak times). Two-way ANOVA was performed on each gene for the factors time (ZT), experimental condition (Exp), and their interaction (ZT×Exp). In addition, post-hoc REGW tests were performed to test significance for each gene in pairwise comparisons of the three experimental conditions, and to define for each experimental condition the time at which each transcript was at its peak. These tests are summarized in Fig. 6 (see Supplementary Table S7 for details).

The response in LT resembled that seen in many previous studies of wild-type Col-0 growing in square-wave light regimes; *LHY* and *CCA1* transcripts peaked around dawn, followed by *PRR9* and *PRR7*, followed by the dusk transcripts (*PRR5*, *TOC1*, and *GI*) and the evening components (*ELF4* and *LUX*), with *ELF3* showing a slightly delayed and weaker oscillation (see the Introduction; see also Flis *et al.*, 2015, 2016 for data collected in square-wave regimes in otherwise similar conditions to those in LT). L^VAR^T and, in particular, L^VAR^T^VAR^ showed modified responses. All genes except *CCA1* and *ELF3* showed a significant effect of Exp, and all genes except *LUX* and *ELF3* showed a highly significant interaction (ZT×Exp), meaning that the peak position and/or amplitude differed significantly between the experimental treatments. Pairwise comparison between LT and L^VAR^T indicated significantly higher expression of *GI* and *LUX* and lower expression for *LHY* and *ELF4* in L^VAR^T (Fig. 6). Pairwise comparison of L^VAR^T^VAR^ with LT or L^VAR^T revealed significantly higher expression of *PRR9* in both comparisons, and significantly lower expression of *LHY*, *PRR5*, *TOC1*, and *ELF4* in both comparisons as well as *GI* and *LUX* in the L^VAR^T comparison (Fig. 6).

Visual inspection of the L^VAR^T^VAR^ time-series reveals significantly prolonged peaks of dawn (*LHY* and *CCA1*) transcript abundance, a significant shift in peak time of *PRR9* transcript, and a pronounced early peak of *GI* transcript at ZT4 in addition to the major peak at ZT10 (Supplementary Fig. S10; Supplementary Table S7). Increased peak abundance for *PRR9*, the pronounced early peak for *GI*, and delayed peak time for *LHY*, *CCA1*, and *PRR9* may be the reason for the divergence of L^VAR^T^VAR^ at ZT4 in the PC analysis plot (Fig. 1C). Overall, these results indicate that large differences between day and night temperatures directly or indirectly restrict *LHY* and *CCA1* expression and promote *PRR9* and *GI* expression.

## Discussion

In a previous study (Annunziata *et al.*, 2017*a*), we analysed diel metabolism in a temperature-controlled glasshouse at the spring equinox on a cloudy day (DLI=7 mol m^−2^ d^−1^) and a sunny day (DLI=12 mol m^−2^ d^−1^) and compared them with sinusoidal simulations at a similar DLI in controlled-environment chambers. We now extend the DLI12 study by adding (i) analyses of metabolites in a variable light and temperature regime, and (ii) analyses of 10 clock transcripts in all three conditions.

### Metabolites and clock transcripts in temperature-controlled regimes with controlled sinusoidal or natural irradiance

The DLI12 comparison came from 2015 when early spring was sunny. On harvest day, the natural light regime differed from the simulated regime by having fluctuating light due to intermittent cloud cover (between 700 µmol m^−2^ s^−1^ and 250 µmol m^−2^ s^−1^ compared with a steady maximum of ~465 µmol m^−2^ s^−1^), and a lower R:FR ratio (1.0 compared with 2.9). The regimes also differed with respect to pre-history; plants in controlled chambers had experienced a recurring regime, whereas irradiance varied from day to day in the glasshouse including automated shading on some days. This may in part explain why biomass was lower in the variable light regime than the sinusoidal light regime. On harvest day, DLI was similar in both regimes.

Compared with the controlled light regime, plants in the natural light regime had more anthocyanin, probably in response to fluctuating irradiance and the resulting intervals of excitation pressure (Steyn *et al.*, 2002). They had lower Gln, some other amino acids, and some organic acids (citrate, isocitrate, and 2OG). The decrease was less marked than in a comparison at a lower irradiance (DLI7; see Annunziata *et al.*, 2017*a*). The DLI7 study was from 2012 when early spring was cloudy with even larger variance in irradiance, possibly explaining the larger decrease in organic acids and amino acids. Annunziata *et al.* (2017*a*) proposed that the low organic acid and amino acid levels in natural light regimes reflect weak buffering of organic acid and N metabolism against a fluctuating environment, compared with the highly sophisticated management of starch reserves (see below for further discussion).

Clock transcripts showed rather similar diel patterns in natural and simulated light regimes. Minor but significant modifications in L^VAR^T included a small pre-dawn rise of *PRR9* transcript, slightly delayed peak time of dawn transcripts, and a slightly earlier rise of *GI* transcript. This may reflect features of the natural regime that are not fully captured in a sinusoidal simulation; for example, light quality or angle. Light induces *PRR9* (Makino *et al.*, 2002; Ito *et al.* 2005, 2007), directly or indirectly regulates *LHY*, *CCA1*, and *GI* expression (Kinmonth-Schutz *et al*., 2013; Staiger *et al.*, 2013), and influences the stability of many clock proteins (Seo and Mas, 2014). Further, PHYTOCHROME B protein interacts with many clock proteins including LHY, CCA1, GI, TOC1, LUX, and ELF3 (Yeom *et al.*, 2014). More generally, our results are consistent with an analysis of a large data set for field-grown rice, which concluded that although many circadian clock transcripts are sensitive to varying irradiation, the threshold is usually at low irradiance (Nagano *et al.*, 2012).

### Diel C and N metabolism in variable compared with controlled temperature regimes

The variable temperature regime differed from the controlled temperature regimes in having lower night temperature (minimum 10–13 °C) and more varied maximum temperatures in the daytime (16–30 °C). In the week preceding harvest, the daily temperature range in the variable regime was 4–20 °C, compared with <4 °C in the controlled temperature regimes. Relatively high irradiance and cool nights prevail around the spring equinox in Golm, when local Arabidopsis accessions proliferate in meadows and disturbed ground.

In the variable temperature regime, starch was degraded slowly and was not completely exhausted by dawn. Starch continued to decline after dawn and, once it started to accumulate (from around ZT4), did so more slowly than in controlled temperature regimes. There were also higher levels of sugars and phosphorylated intermediates around dawn and for much of the light period in L^VAR^T^VAR^ than in the temperature-controlled regimes.

The high starch and sugars at dawn may be partly due to low night temperature slowing down C utilization for growth. However, Pyl *et al.* (2012) reported that starch was completely exhausted at night temperatures as low as 12 °C. Incomplete exhaustion of starch in our current study may be partly due to irradiance being much higher than in Pyl *et al.* (2012). In addition, as will be discussed later, incomplete mobilization of starch at dawn and continued mobilization after dawn was paralleled by a delay in peak time for *LHY* and *CCA1* transcripts.

Slow starch accumulation in the light, despite high levels of sugars and phosphorylated metabolites, may be an indirect consequence of the cool night; it is known that starch accumulation is slower when starch is not fully exhausted in the preceding 24 h cycle (Gibon *et al.*, 2004; Mugford *et al.*, 2014; Mengin *et al.*, 2017). However, there may also be more direct effects, as low temperature is known to shift C allocation away from starch towards accumulation of sucrose and other cryoprotectants (Guy, 1990; Guy *et al.*, 1992; Strand *et al.*, 1999; Stitt and Hurry, 2002; Obata and Fernie, 2012).

Variable temperature had a larger impact on N metabolism than on C metabolism. Amino acid levels were high in the natural light and variable temperature regime compared with the temperature-controlled sinusoidal simulation and, in particular, the temperature-controlled natural light regime. The high levels were not due to a shortfall in C; sugars, phosphorylated intermediates, and most organic acids including 2OG were higher in the variable temperature than the temperature-controlled natural light regime (see previous section), and analyses of reporter transcripts did not provide any evidence for activation of C starvation signalling (Supplementary Fig. S8). The increase of many minor amino acids around dusk (Supplementary Fig. S4D, E) coincided with a drop in temperature (Supplementary Fig. S1) that is likely to restrict protein synthesis (Guy 1990; Usadel *et al.*, 2008*b*). Decreased use of amino acids for protein synthesis may also contribute to the elevated levels of most major amino acids at the end of the night (Supplementary Fig. S4C).

Taken together, our results indicate that fluctuating light impairs N metabolism, and that this is reversed by low night temperature. Irradiance and temperature typically change together in natural environments. As a result, any shortfall in reserve formation due to varying light in the daytime may be buffered by decreased consumption during the cool night. It can be speculated that plants have evolved regulatory mechanisms that deal robustly with combined changes of irradiance and temperature, but not with changes of irradiance alone. Similar reasoning has been used to explain the close connections between light and temperature signalling in many physiological and developmental responses (Franklin *et al.*, 2014). It is possible that harmful effects of warm nights on crop yield (Peng *et al.*, 2004; Lobell *et al.*, 2011) may sometimes be due to C and N metabolism becoming imbalanced when the buffering effects of low night temperature are removed. However, in rice, high night temperature (28 °C) is associated with high organic acids and amino acids (Glaubitz *et al.*, 2015, 2017), whereas in our study these metabolites were high in a regime with cool nights. This may reflect species differences, or differing responses in the high and low temperature range.

Biomass in the variable light and temperature regime was marginally higher than in the controlled light and temperature regime, and significantly higher than in the variable light and controlled temperature regime (Supplementary Fig. S2). As already discussed, the latter may be low due to shading on some days. Three factors may explain why biomass accumulation is maintained in the variable temperature regime, despite the plants containing higher levels of starch and many metabolites. First, the amount of C accumulated in metabolites is relatively low compared with rosette biomass. Secondly, low night temperature may reduce respiration and partly compensate for accumulation of C in starch and other metabolites. Thirdly, a higher protein content (Fig. 2E) might allow more efficient light utilization, especially during periods of high irradiance in natural regimes. However, we point out that our experiment was designed to investigate the impact of controlled versus variable conditions on metabolite levels and clock transcript abundance on harvest day. Biomass comparisons are more difficult, because biomass depends on climatic conditions over the entire life history, and these varied from day to day in a treatment-dependent manner.

### Fluctuating conditions decrease metabolic connectivity

Diel changes of metabolites were highly co-ordinated in a controlled environment, as seen in many earlier studies. Not only carbohydrates but also organic acids and amino acids typically show rather co-ordinated responses (Noctor *et al.*, 2002; Fritz *et al.*, 2006; Woodrow *et al.*, 2017). Most metabolites increased in the daytime and declined during the night, reflecting the build-up of reserves in the light and their consumption in the dark. Citrate, isocitrate, and aconitate showed a reverse pattern, as seen previously (Urbanczyk-Wochniak *et al.*, 2005; Sulpice *et al.*, 2014). In the light, mitochondrial pyruvate dehydrogenase is inhibited (Tovar-Méndez *et al.*, 2003) and this restricts movement of newly fixed C into citrate (Szecowka *et al.*, 2013; Ishihara *et al.*, 2015; Figueroa *et al.*, 2016). The citrate that is accumulated at night provides a supply of 2OG in the light to support *de novo* ammonium assimilation and reassimilation of photorespiratory ammonium (Tcherkez *et al.*, 2012*a*, *b*; Ishihara *et al.*, 2015).

Fluctuating light led to a dramatic decrease in metabolic connectivity, affecting amino acids in particular. This may in part reflect the smaller rise of amino acid levels in the light period in the variable temperature regime. Variable temperature did not lead to additional loss of connectivity, even though there were large differences in the diel timing of changes in metabolite levels and the absolute levels of metabolites compared with the temperature-controlled regimes. These changes in connectivity are consistent with the idea that plants have evolved regulatory networks to cope with an environment where irradiance and temperature often vary in a co-ordinated manner.

### Variable temperature in the ambient range triggers low temperature responses

Some sugars and amino acids, and their derivatives, act as compatible solutes (Ristic and Ashworth, 1993; McKown *et al.*, 1996; Xin and Browse, 1998). Variable temperature led to accumulation of such metabolites. For example, Pro was 10 times higher in variable compared with controlled temperature conditions. Accumulation of compatible solutes, like many other low temperature responses, is under the control of C-REPEAT BINDING FACTOR (CBF) transcription factors (Thomashow, 2010). Although usually studied in the context of cold acclimation and frost tolerance, CBFs are induced by moderate decreases in temperature and operate as a rheostat, allowing plants to respond and adjust over wide temperature ranges (Zarka *et al.*, 2003; Vogel *et al.*, 2005; Usadel *et al.*, 2008*a*).

There was a remarkable 4- to 8-fold higher rosette protein content in the variable temperature compared with controlled temperature regimes. Leaves that develop at low temperatures have a high protein content (Stitt and Hurry, 2002; Usadel *et al.*, 2008*a*). The protein content in our variable light and temperature regime is as high as or higher than that in plants growing at 4 °C. This may be partly due to increased ribosome content. Low temperature increases expression of ribosomal proteins in a *CBF*-independent manner, with this already being significant at 14 °C (Provart *et al.*, 2003; Usadel *et al.*, 2008*a*). Ribosomal protein expression will also be promoted by high sugar levels in the variable temperature regime (Usadel *et al.*, 2008*b*). Interestingly, when Arabidopsis was grown in square-wave regimes, low night temperature did not lead to an increase in protein content when daytime temperature was high (Pyl *et al.*, 2012). This indicates that the high protein content in our study may be due to low temperature early in the light period. However, it may also be due to co-variation of temperature and light. During diel cycles, protein synthesis is faster in the light (Ishihara *et al.*, 2015, 2017) and leaf expansion is faster at night (Apelt *et al.*, 2015, 2017). The quasi-parallel changes in irradiance and temperature that occur in natural or near-natural environments may favour protein synthesis and restrict expansion growth. In addition, it would be interesting to investigate if an interaction between light and temperature signalling (Casal, 2012, 2013; Franklin *et al.*, 2014) contributes to the increase in protein.

Anthocyanin accumulated to high levels in the variable temperature regime. Suboptimal temperature amplifies the impact of high light (Nozzolillo *et al.*, 1990; Leng *et al.*, 2000; Ilk *et al.*, 2015). Maximal anthocyanin accumulation requires cool nights (10 °C) followed by mild daytime temperatures (25 °C); low temperature enhances transcription of regulatory and biosynthetic genes, whilst post-translational events leading to anthocyanin synthesis require higher temperatures (Christie *et al.*, 1994). In addition, sugars induce anthocyanin synthesis. This resembles the explanation for the high protein content (see above).

### Diel clock transcript abundance in variable and controlled temperature regimes

The Arabidopsis clock is temperature compensated and maintains a near 24 h period with large amplitudes in transcript abundance at temperatures between 12 °C and 27 °C (Gould *et al.*, 2013). However, most previous studies of the impact of temperature on the clock used continuous light or recurring square-wave light–dark cycles. We have investigated the impact of temperature against the background of varying irradiance. PCA revealed that whereas the diel changes in variable temperature and controlled temperature regimes were separated for metabolites, they overlapped for clock transcript abundance. This adds to the evidence that temperature compensation renders the clock robust against varying temperature.

Nevertheless, there were divergences between the PC trajectories in the variable and controlled regimes at ZT4 and an opposite divergence at ZT6–ZT10. Differences at ZT4 included a delay in peak time for *LHY*, *CCA1*, and *PRR9* (from about ZT2 to ZT4), reduced *LHY* and *CCA1* abundance, increased *PRR9* abundance, and a large early peak of *GI* at ZT4. Previous studies in Arabidopsis reported an increase of *PRR9* transcript at both low (Bieniawska *et al.*, 2008) and high temperature (Gould *et al.*, 2013), pointing to strong temperature sensitivity for this clock component. A minor early peak of *GI* transcript was reported in some earlier studies (Locke *et al.*, 2005; Paltiel *et al.*, 2006; Edwards *et al.*, 2010; Flis *et al.*, 2016). In our variable temperature regime, this early peak is higher than the peak at ZT8–ZT10. Divergence in the PC plot at ZT6–ZT10 may reflect lower transcript abundance for dusk and evening components such as *PRR5*, *TOC1*, and *ELF4*.

Thus, our data indicate that low night temperature directly or indirectly restricts *LHY* and *CCA1* expression and promotes *PRR9* and *GI* expression in the first hours of the light period. Temperature interacts closely with light signalling via phytochrome, with low temperatures slowing thermal reversion of the active (Pfr) to the inactive (Pr) form, which will sensitize the fluence response of phytochrome signalling at a given Pfr/Pr ratio (Jung *et al.*, 2016; Legris *et al.*, 2016; Delker *et al.*, 2017). Pfr acts positively on expression of many clock transcripts (see above). This might contribute to the elevated peak of *PRR9* transcript. The early *GI* peak is very sensitive to light, being induced in a gated manner by blue light acting via cryptochrome signalling (Paltiel *et al.*, 2006). However, light signalling would be expected to advance rather than delay peak time of the dawn transcripts, indicating that further factors in the variable temperature regime negatively regulate *LHY* and *CCA1*. Possibilities include repression by *PRR9* which, together with *PRR7*, has been shown to contribute to the temperature compensation of *LHY* and *CCA1* in constant temperature regimes (Salomé *et al.*, 2010). The robustness of dusk and evening transcripts to low night temperature might be partly explained by their lower abundance at dusk in the variable temperature regime. This might aid their decay by self-inhibition (Pokhilko *et al.*, 2012; Fogelmark and Troein, 2014), protein destabilization (Seo and Mas, 2014), and other forms of temperature compensation. Interestingly, peak *LUX* transcript abundance was not decreased, confirming an earlier study at 4 °C (Bieniawska *et al.*, 2008). The large oscillations of *LUX* at low temperature are due to transcriptional regulation by *CBF1* (Chow *et al.*, 2014).

The ~2 h phase delay of the dawn components in the variable temperature compared with temperature-controlled regimes may delay dawn clock activity until the temperature rises far enough for rapid metabolism and photosynthesis to resume. For example, the arithmetic division model (Scialdone *et al.*, 2013) proposes that the rate of starch degradation is paced to exhaust starch by dawn as anticipated by the clock. The phase delay of the dawn genes might contribute to the slower and incomplete mobilization of starch in the variable temperature regime (Fig. 2A).

Our results can be compared with the findings of a large study with rice grown in the field during the summer in Japan (Nagano *et al.*, 2012; Matsuzaki *et al.*, 2015). This study also found that the diel dynamics of transcripts, including clock transcripts, were more sensitive to day to day variation in temperature than irradiance. However, in rice, the changes in clock transcripts were mainly due to higher values during the trough at night, whereas in our study the changes were more marked in the light period. This may reflect species differences, or the lower night temperatures in our study.

Finally, this study and that of Annunziata *et al.* (2017*a*) underline that results obtained in recurring environmental conditions in climate chambers may fail to capture responses that are important in field conditions, and that studies of the impact of single varying environmental parameters may fail to capture the complexity of interactions between multiple simultaneously varying or co-varying factors. The combinatorial explosion of multiple varying factors creates a huge experimental space, and strategies are needed to prioritize which factors and combinations of them should be studied. One first step may be to profile well-understood processes in different field or near-field conditions, in order to identify responses that differ from those seen in controlled conditions, link the novel responses to particular environmental variables (see, for example, Nagano *et al.*, 2012; Matsuzaki *et al.*, 2015), and formulate hypotheses about the reason for the novel response. The next and most challenging step will be to reconstruct near-field environments in climate chambers and generate a reproducible experimental system in which these hypotheses can be tested.

## Supplementary data

Supplementary data are available at *JXB* online.

**Fig. S1.** Diel irradiance and temperature in the three growth regimes.

**Fig. S2.** Plant morphology on the day of harvest, fresh and dry weight, and water content.

**Fig. S3.** Principal component analysis of core circadian clock gene data from variable light- and variable temperature-grown Arabidopsis plants (supporting information to Fig. 1).

**Fig. S4.** Diel profiles of metabolites in different growth regimes (supporting information to Fig. 2).

**Fig. S5.** Diel time series of metabolite levels compared between growth regimes (supporting information to Fig. 3).

**Fig. S6.** Diel time series of different metabolites compared within a given growth regime (supporting information to Fig. 5).

**Fig. S7.** Correlation between Tre6P and sucrose in different growth regimes.

**Fig. S8.** Transcript abundance for C starvation marker genes.

**Fig. S9.** Log scale plots of diel transcript abundance of core circadian clock genes (supporting information to Fig. 6).

**Fig. S10.** Radar plots of transcript abundance of core circadian clock genes (supporting information to Fig. 6).

**Table S1.** Climate data.

**Table S2.** Primer sequences and detailed description of the qRT-PCR procedure.

**Table S3.** Fresh and dry weight and water content measured on the day of harvest.

**Table S4.** Metabolite data (original data for Figs 1A, 2; Supplementary Fig. S4).

**Table S5.** Core clock genes and C-starvation marker genes (original data for Figs 1C, 6; Supplementary Figs S8, S9, S10).

**Table S6.** Statistical analysis performed on the metabolites shown in Supplementary Fig S4.

**Table S7.** Effect of experimental condition and sampling time on abundance of circadian clock transcripts as determined by two-way ANOVA followed by a post-hoc Ryan–Einot–Gabriel–Welsh (REGW) test (supporting information to Fig. 6).

## Supplementary Material

Supplementary Table S1

Supplementary Table S2

Supplementary Table S3

Supplementary Table S4

Supplementary Table S5

Supplementary Table S6

Supplementary Table S7

Supplementary Figures S1-S10
