# Supplementary material for: Response of Arabidopsis primary metabolism and circadian clock to low night temperature in a natural light environment
Source: J Exp Bot. 2018 Jul 25;69(20):4881–95. doi: 10.1093/jxb/ery276 (PMC6137998; doi:10.1093/jxb/ery276)
Supplement: Supplementary Table S1 [file ery276_suppl_supplementary_table_s1.pdf]

Supplemental Table S1. Climate data for the growth cycle in $L^{VAR}T$ and $L^{VAR}T^{VAR}$ conditions. Temperature measurements in °C: Min T=minimum temperature; Max T=maximum temperature; T range=temperature range; Heat sum=thermal sum calculated from 10. March onwards as the cumulative sum of (min T + max T)/2.
DLI=Daily Light Integral (mol m^{-2} d^{-1}).

Growth condition	Date	Min T (°C)	Max T (°C)	T range (°C)	Heat Sum (°C)	DLI (mol m^{-2} d^{-1})
$L^{VAR}T^{VAR}$	10MAR2015	12.0	20.0	8.0	16.0	8
$L^{VAR}T^{VAR}$	11MAR2015	11.0	19.0	8.0	31.0	6
$L^{VAR}T^{VAR}$	12MAR2015	11.0	16.0	5.0	44.5	3.8
$L^{VAR}T^{VAR}$	13MAR2015	11.0	19.0	7.0	59.5	8.9
$L^{VAR}T^{VAR}$	14MAR2015	11.0	17.0	6.0	73.5	4.9
$L^{VAR}T^{VAR}$	15MAR2015	11.0	18.0	7.0	88.0	4.6
$L^{VAR}T^{VAR}$	16MAR2015	12.0	27.0	15.0	107.5	15.9
$L^{VAR}T^{VAR}$	17MAR2015	11.0	30.0	19.0	128.0	16.7
$L^{VAR}T^{VAR}$	18MAR2015	13.0	24.0	11.0	146.5	19.9
$L^{VAR}T^{VAR}$	19MAR2015	12.0	24.0	12.0	164.5	19.6
$L^{VAR}T^{VAR}$	20MAR2015	12.0	22.0	10.0	181.5	17.2
$L^{VAR}T^{VAR}$	21MAR2015	12.0	16.0	4.0	195.5	5.6
$L^{VAR}T^{VAR}$	22MAR2015	10.0	25.0	15.0	213.0	21.4
$L^{VAR}T^{VAR}$	23MAR2015	10.0	30.0	20.0	233.0	20.9
$L^{VAR}T^{VAR}$	24MAR2015	12.0	27.0	15.0	252.5	14.2

Growth condition	Date	Min T (°C)	Max T (°C)	T range (°C)	Heat Sum (°C)	DLI (mol m^{-2} d^{-1})
$L^{VAR}T$	10MAR2015	19.8	23.2	3.4	21.5	7.6
$L^{VAR}T$	11MAR2015	19.7	23.4	3.7	43.0	5.3
$L^{VAR}T$	12MAR2015	19.7	23.0	3.3	64.0	3.5
$L^{VAR}T$	13MAR2015	19.8	23.3	3.5	86.0	7.5
$L^{VAR}T$	14MAR2015	19.8	22.9	3.1	107.0	4.4
$L^{VAR}T$	15MAR2015	19.7	22.9	3.2	128.5	3.9
$L^{VAR}T$	16MAR2015	19.7	23.7	4.0	150.0	12.6
$L^{VAR}T$	17MAR2015	19.8	23.6	3.8	172.0	12.5
$L^{VAR}T$	18MAR2015	19.8	23.9	4.1	194.0	10.7
$L^{VAR}T$	19MAR2015	19.7	23.9	4.2	216.0	11.3
$L^{VAR}T$	20MAR2015	19.7	23.9	4.2	237.0	8.8
$L^{VAR}T$	21MAR2015	19.8	23.3	3.5	259.0	5
$L^{VAR}T$	22MAR2015	19.7	23.9	4.2	281.0	12
$L^{VAR}T$	23MAR2015	19.8	23.9	4.1	303.0	11.6
$L^{VAR}T$	24MAR2015	19.8	23.3	3.5	324.0	11.7
