# Supplementary material for: Response of Arabidopsis primary metabolism and circadian clock to low night temperature in a natural light environment
Source: J Exp Bot. 2018 Jul 25;69(20):4881–95. doi: 10.1093/jxb/ery276 (PMC6137998; doi:10.1093/jxb/ery276)
Supplement: Supplementary Table S2 [file ery276_suppl_supplementary_table_s2.pdf]

Supplemental Table S2. Primer sequences and detailed description of the qRT-PCR procedure.

AGI code	Primer ID	Primer sequence (5'->3')
At1g134401	GAPDH-5'-F<br>GAPDH-5'-R	TAACGTGGCCAAAATGATGC<br>GTTCTCCACAACCGCTTGGT
At1g134401	GAPDH-3'-F<br>GAPDH-3'-R	TTGGTGACAACAGGTCAAGCA<br>AAACTTGTCGCTCAATGCAATC
At2g28390	SAND-F<br>SAND-R	AACTCTATGCAGCATTTGATCCACT<br>TGATTGCATATCTTTATCGCCATC
At1g13320	PDF2-F<br>PDF2-R	TAACGTGGCCAAAATGATGC<br>GTTCTCCACAACCGCTTGGT
At5g60390	EF-1α-F<br>EF-1α-R	TGAGCACGCTCTTCTTGCTTTCA<br>GGTGGTGGCATCCATCTTGTTACA
At4g05320	UBQ10-F<br>UBQ10-R	GGCCTTGTATAATCCCTGATGAATAAG<br>AAAGAGATAACAGGAACGGAAACATAGT
At3g18780	ACT2-F<br>ACT2-R	CTTGCACCAAGCAGCATGAA<br>CCGATCCAGACACTGTACTTCCTT
At5g08290	YLS8-F<br>YLS8-R	TTACTGTTTCGGTTGTTCTCCATTT<br>CACTGAATCATGTTCGAAGCAAGT
At2g28000	CPN60ALPHA1-F<br>CPN60ALPHA1-R	TATCGAGGAAGGCATAGTTCCAGGTGGTGG<br>CGGGAATAACAGTGGAGAGATGCACCAAAG
At3g13470	CPN60BETA2-F<br>CPN60BETA2-R	CCGACAAAGGTTGTGAGAT<br>TACCAGCAGGAACTGGCTCT
At1g10070	BCAT2-F<br>BCAT2-R	ACCGGGGATGAATCTGTCT<br>TCTGTGACCCATCCCTTGTT
At2g45170	ATG8e-F<br>ATG8e-R	TGTGATTCGGAAGAGAATCAAACTAAGTGC<br>GCCATCTTCGTCTTTCTTATCCTCGTAAAC
At1g01060	LHY-F<br>LHY-R	TGCCTCAAAGCTTTTCGCCTCCT<br>GTCTGCAGCACAAGAATCCTGGCT
At2g46830	CCA1-F<br>CCA1-R	TCCAATGCACGCCGCAGTAGAA<br>AGGCAATTCGACCCTCGTCAGACA
At2g46790	PRR9-F<br>PRR9-R	AGCTAGCAGAACAACGTCCTCGAGT<br>CGTCTGAATTCACGGTTCGCACGA
At5g02810	PRR7-F<br>PRR7-R	AGAGGTGCTTCCGAAAGAAGGTACGA<br>ACGCACAAATTGGCCTCGCACT
At5g24470	PRR5-F<br>PRR5-R	TGCAATGGCTCCTGCTTCACTCTC<br>ACTGTACTCATGCGGGCTAACGGA
At5g61380	TOC1-F<br>TOC1-R	TGATGGATCGGGTTTCTCTGCACCA<br>TGAGGCATCATGGCTGCTGATTGC
At1g22770	GI-F<br>GI-R	TGCGGGCAACTGATGGAATGCT<br>TGCTCTTGCCGTGGCTTCAAGT
At3g46640	LUX-F<br>LUX-R	CAGCGGTAATGTTGGAGTGCCGAT<br>TGGCATCTGCATCATCTGTTGCGT
At2g40080	ELF4-F<br>ELF4-R	AGTTTCTCGTCGGGCTTTCACGGT<br>TAAGCTCTAGTTCCGGCAGCACCA
At2g25930	ELF3-F<br>ELF3-R	ACAACAAGAGATGGGGGAGGAGTGAC<br>ACTCGCGAGCTTTGCGTTGTGA

Abbreviations of reference genes: *GLYCERALDEHYDE-3-PHOSPHATE DEHYDROGENASE* (GAPDH, At1g13440), *SAND FAMILY PROTEIN* (*SAND*, At2g28390), *PROTEIN PHOSPHATASE 2A* (*PDF2*, At1g13320), elongation factor-1α *ELONGATION FACTOR-1A* (*EF-1α*, At5g60390), *UBIQUITIN10* (*UBQ10*, At4g05320), *ACTIN2* (*ACT2*, At3g18780), and *MITOSIS PROTEIN YLS8* (*YLS8,* At5g08290).

Abbreviations of C starvation marker genes: *CHAPERONIN-60ALPHA1* (*CPN60ALPHA1*, At2g28000); *CHAPERONIN-60BETA2* (*CPN60BETA2*, At3g13470); *BRANCHED-CHAIN-AMINO-ACID AMINOTRANSFERASE 2 (BCAT2, At1g10070); AUTOPHAGY-RELATED PROTEIN 8e* (*ATG8e,* At2g45170).

Abbreviations of circadian clock genes: *LATE ELONGATED HYPOCOTYL* (*LHY,* At1g01060); *CIRCADIAN CLOCK ASSOCIATED1* (*CCA1,* At2g46830); *PSEUDO-RESPONSE REGULATOR9, 7 and 5* (*PRR9,* At2g46790*; PRR7*, At5g02810; *PRR5,* At5g24470); *TIMING OF CAB EXPRESSION1* (*TOC1,* At5g61380); *GIGANTEA* (*GI,* At1g22770); *LUX ARRHYTHMO* (*LUX,* At3g46640); *EARLY FLOWERING4* and *3* (*ELF4,* At2g40080; *ELF3,* At2g25930).

RNA extraction and qRT-PCR: Total RNA was isolated from 50-100 mg of homogenized tissue using the RNeasy Plant mini Kit (Qiagen, Hilden, Germany) according to manufacturer's instructions. RNA concentration and integrity was measured using a NanoDrop ND-1000 UV-Vis spectrophotometer (Nano- Drop Technologies, Böblingen, Germany) before and after DNase I digestion (Turbo DNA-free DNase I; Applied Biosystems/Ambion, Darmstadt, Germany). Absence of genomic DNA contamination in DNase I-treated samples was tested by PCR using primers (5′-TTTTTTGCCCCCTTCGAATC-3′ and 5′-ATCTTCCGCCACCACATTGTAC-3′) designed to amplify an intron sequence of a reference gene (At5g65080). cDNA was synthesized from the DNase digested RNA (50 µl) using SuperScript™IV Reverse Transcriptase (Invitrogen/Life Technologies, Darmstadt, Germany). RNA, dNTP and oligo (dT)18 primer were denaturated at 65 °C for 5 min, followed by incubation on ice for at least 1 min prior to adding the remaining reagents. cDNA synthesis was carried out at 50 °C for 50 min, then at 55 °C for 15 min, before being deactivated by heating at 70 °C for 15 min. The relative cDNA concentration and quality were determined using primers against *Ubiquitin10* (*UBQ10*, At4g05320) (Czechowski *et al*., 2005) and primers against the 3´ and 5´ regions of *Glyceraldehyde-3-phosphate dehydrogenase* (*GAPDH*, At1g13440). Samples with Ct 5´GAPDH - Ct 3´GAPDH values > 2 were excluded from further analyses.

qRT-PCR was performed on an ABI Prism® 7900 HT real time PCR system (Applied Biosystems/Life Technologies, Darmstadt, Germany) in 384 well PCR plates with a total reaction volume of 10 µl (4 µl forward and reverse primer mixture (0.5 µM, each), 1 µl cDNA and 5 µl Power SYBR® Green-PCR Master Mix (Applied Biosystems/Life Technologies, Darmstadt, Germany) using the following program: 50 °C for 2 min, 95 °C for 10 min, 40 cycles of 95 °C for 15 sec and 60 °C for 60 sec, followed by a denaturation step of 95 °C for 15 sec, 60 °C for 15 sec with a continuous temperature increase (0.3 °C/s) to 95 °C for 15 sec. Steps 5, 6 and 7 were introduced to record a dissociation or melting curve of each product in order to detect non-specific amplification. qRT-PCR was performed on two biological replicates with three technical replicates each to monitor the transcript levels of 10 core circadian clock genes (*LHY*, *CCA1*, *PRR9*, *PRR7*, *PRR5*, *TOC1*, *GI*, *LUX*, *ELF4* and *ELF3*). Four carbon-starvation marker genes were also analyzed: *Chaperonin-60 alpha1*, (*CPN60ALPHA1*, At2g28000), *Chaperonin-60beta2* (*CPN60BETA2*, At3g13470), *Branched-chain-amino-acid aminotransferase 2* (*BCAT*-2, At1g10070), and *Autophagy-related protein 8e* (*ATG8e*, At2g45170).

For relative transcript quantification, *GAPDH, SAND family protein* (*SAND*, At2g28390), *Protein phosphatase 2A* (*PDF2*, At1g13320), *Elongation factor-1α* (*EF-1α*, At5g60390), *UBQ10*, *Actin 2* (*ACT2*, At3g18780), and *Mitosis protein YLS8* (*YLS8*, At5g08290) genes were used as reference genes (reference gene index, RGI). Expression values and data analyses were as follows: Ct values across all reference genes were geometrically averaged to build the reference gene index (RGI), subtracted from the Ct values of the different tested genes (ΔCt) and relative expression was then calculated as $2^{-\Delta Ct}$ for each sample. Average and standard deviation were calculated for each time point. Primers (listed in the Suppl. Table S2) were designed using the PRIME program of GCG Wisconsin Package version 10.2 and synthesized at MWG Biotech AG (Ebersberg, Germany).
