# Supplementary material for: Response of Arabidopsis primary metabolism and circadian clock to low night temperature in a natural light environment
Source: J Exp Bot. 2018 Jul 25;69(20):4881–95. doi: 10.1093/jxb/ery276 (PMC6137998; doi:10.1093/jxb/ery276)
Supplement: Supplementary Table S7 [file ery276_suppl_supplementary_table_s7.pdf]

Supplemental Table S7. Effect of experimental condition (LT, $L^{VAR}T$, $L^{VAR}T^{VAR}$) and sampling time (ZT) on abundance of circadian clock transcripts as determined by two-way analysis of variance (ANOVA) followed by a *post hoc* Ryan-Einot-Gabriel-Welsh (REGW) test (supporting information to Fig. 6).

The tests were performed to (i) determine, for each gene, statistically significant pairwise differences between experimental conditions and (ii) to identify peak time for each transcript and treatment.

***p*-value in two-way analysis of variance (ANOVA) on factors (F-test (ANOVA))**: Two-way-ANOVA (proc GLM, SAS 9.4) was performed on each gene for the factors sampling time (ZT), experimental condition (Exp), and their interaction effect (ZTxExp). As expected, all clock genes had a highly significant effect of ZT. All genes except *CCA1* and *ELF3* showed a significant effect for Exp. All genes except *LUX* and *ELF*3 also showed a highly significant interaction effect (ZTxExp), which means that the peak position and/or amplitude differed between the experimental treatments.

Classification in REGW Procedure (Exp): For each gene, we also performed a pairwise comparison applying the REGW procedure (alpha = 0.05) for the means of the factor Exp. This analysis orders, for a given transcript, the mean values for abundance from lowest to highest and tests them for significant differences and, if significant, repeats this procedure for the next lowest and highest mean value. It groups experimental treatments based on significant changes in abundance for that transcript. Experimental treatments with non-significant differences in abundance of a given transcript are denoted by the same letter (by default, 'a') and experimental treatments with significantly different abundances of a given transcript receive different letters, with 'a' denoting the treatment with the highest abundance, and 'b' and (where all three treatments are significantly different) 'c' denoting treatments with progressively lower abundance. For most transcripts with a significant effect of Exp (*LHY*, *PRR5*, *TOC1*, *GI*, *LUX* and *ELF4*), transcript abundance is significantly lower in $L^{VAR}T^{VAR}$ than in $L^{VAR}T$. For *PRR9* transcript abundance is significantly higher in $L^{VAR}T^{VAR}$ than in both LT and $L^{VAR}T$.

Classification in REGW Procedure (ZT): To identify the time at which transcript abundance peaked for each gene and experiment, we performed a pairwise REGW procedure (alpha = 0.05) for the means of the factor ZT from ZT0 - ZT18. The estimate peak times indicate, e.g., a delay of *PRR9* transcript peak from ZT2 ($L^{VAR}T$) to ZT4 ($L^{VAR}T^{VAR}$). The analysis is not shown for *ELF3,* because the peak was very broad in all three treatments.

	***p*-value in F-test (ANOVA)**			**Classification in REGW Procedure (Exp)**			**Classification in REGW Procedure (ZT)**		
Gene	ZT	Exp	ZTxExp	LT	$L^{VAR}T$	$L^{VAR}T^{VAR}$	Highest ZT (LT)	Highest ZT ($L^{VAR}T$)	Highest ZT ($L^{VAR}T^{VAR}$)
LHY	0.0001	0.0003	0.0001	**a**	**b**	**c**	0; 2	2	0; 2; 4
CCA1	0.0001	0.6752	0.0001	a	a	a	0; 2	0; 2	0; 2; 4
PRR9	0.0001	0.0084	0.0001	**b**	**b**	**a**	2; 4	2	4
PRR7	0.0001	0.0003	0.0001	a	a	a	6; 8;10	2; 4; 6; 8	4; 6
PRR5	0.0001	0.0214	0.0002	**a**	**a**	**b**	8	8	8; 10
TOC1	0.0001	0.0013	0.0001	**a**	**a**	**b**	10	10	10
LUX	0.0001	0.0256	0.1240	**b**	**a**	**b**	10	10	10
GI	0.0001	0.0082	0.0001	**b**	**a**	**b**	8; 10	8; 10	4; 10
ELF4	0.0001	0.0001	0.0001	**a**	**b**	**c**	10	10	10
ELF3	0.0001	0.1695	0.0834	a	a	a			
