# Supplementary material for: Response of Arabidopsis primary metabolism and circadian clock to low night temperature in a natural light environment
Source: J Exp Bot. 2018 Jul 25;69(20):4881–95. doi: 10.1093/jxb/ery276 (PMC6137998; doi:10.1093/jxb/ery276)
Supplement: Supplementary Figures S1-S10 [file ery276_suppl_supplementary_figures_s1-s10.pdf]

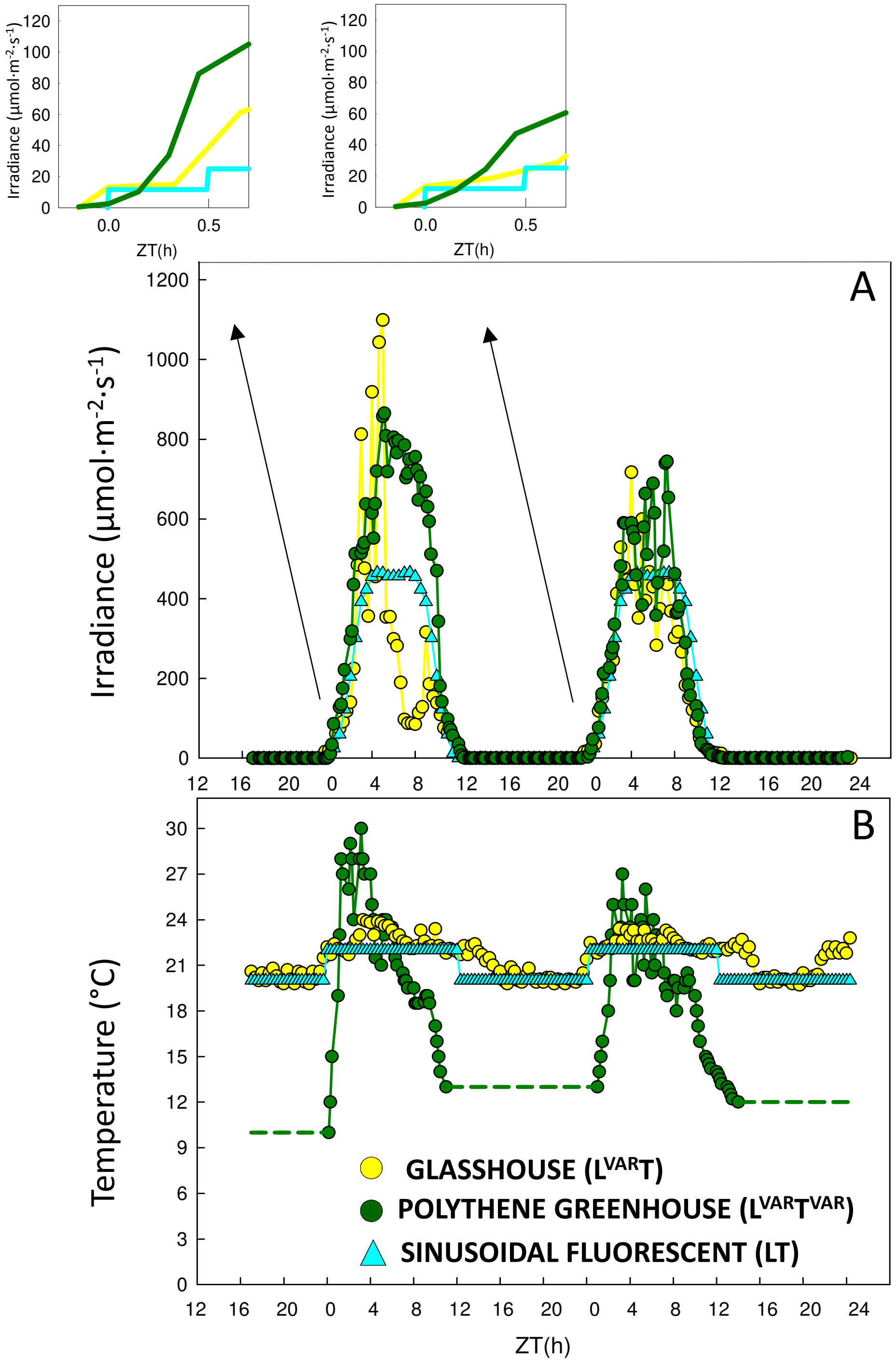

Supplemental Fig. S1. Diel irradiance and temperature in the three growth regimes: (i) controlled light and temperature (LT); (ii) variable light and controlled temperature ($L^{VAR}T$) and (iii) variable light and variable temperature ($L^{VAR}T^{VAR}$). Time series are aligned to the time of first light (LT) or the time of sunrise in Golm ($L^{VAR}T$, $L^{VAR}T^{VAR}$). (A) Irradiance was measured at 20-min intervals in a naturally illuminated glasshouse ($L^{VAR}T$, yellow circles) and at 15-min intervals in a naturally illuminated polythene greenhouse ($L^{VAR}T^{VAR}$, green circles) on the day of harvest and on the preceding day around the vernal equinox in 2015. On the harvesting day plants received an irradiance with a daily light integral (DLI) of ~12 mol m^{-2} d^{-1}. Plants were also grown in controlled environment chambers with a 12-h photoperiod and similar DLI of ~12 mol m^{-2} d^{-1}. Illumination was provided by white fluorescent tubes with sinusoidal light profiles (LT, cyan triangles) and the irradiance was measured at 30-min intervals. The inserts show the change in irradiance around dawn on an expanded scale on the preceding dawn and on the day of harvest. Dawn was sunnier on the preceding day. The sinusoidal simulation was designed to match more closely the harvest day. Light increased slightly more rapidly in the polythene greenhouse than the glasshouse due to some shading by internal structures. With the alignment shown, first light came earlier in the natural regimes than the sinusoidal simulation, the first irradiance in the simulation (~11 µmol·m^{-2}·s^{-1}) transiently exceeded the irradiance in the natural regimes. From 10 min after ZT0, the irradiance in the natural regimes exceeded that in the sinusoidal simulation. (B) Temperature was measured every 20 minutes on the day of harvest in the glasshouse, polythene greenhouse (yellow and green circles, respectively) and growth chamber (cyan triangles). ZT, zeitgeber time (hours after dawn). Frost protection heating was activated at a threshold of about 10°C (dotted line).

	**GLASSHOUSE ($L^{VAR}T$)**	**POLYTHENE GREENHOUSE ($L^{VAR}T^{VAR}$)**	**SINUSOIDAL FLUORESCENT (LT)**
**Morphology**	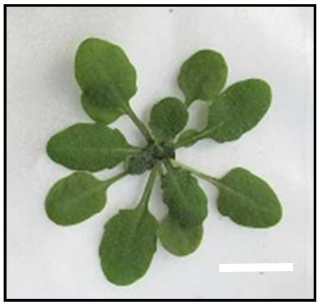	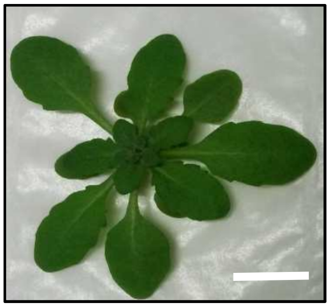	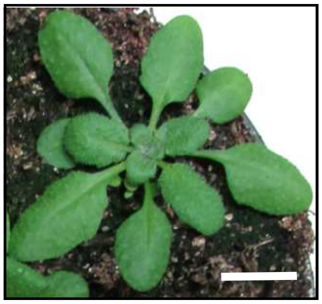
**Fresh weight (g per rosette)**	0.106±0.022	0.178±0.03	0.133±0.036
**Dry weight (g per rosette)**	0.010±0.003	0.019±0.003	0.017±0.007
**Water content (%)**	90.6 ±0.78	88.7±1.16	87.4 ±2.89

Supplemental Fig. S2. Plant morphology on the day of harvest, fresh and dry weight and water content. Arabidopsis Col-0 plants were grown in a naturally illuminated and temperature controlled glasshouse ($L^{VAR}T$) and a naturally illuminated polythene greenhouse with less controlled temperature ($L^{VAR}T^{VAR}$) around the vernal equinox (March 2015), and under an artificial light regime with controlled light and temperature (LT) with a 12-h photoperiod. Artificial illumination was provided by white fluorescent lamps with a sinusoidal profile. Four-week-old plants were harvested at the end of the day. White bar=1cm.

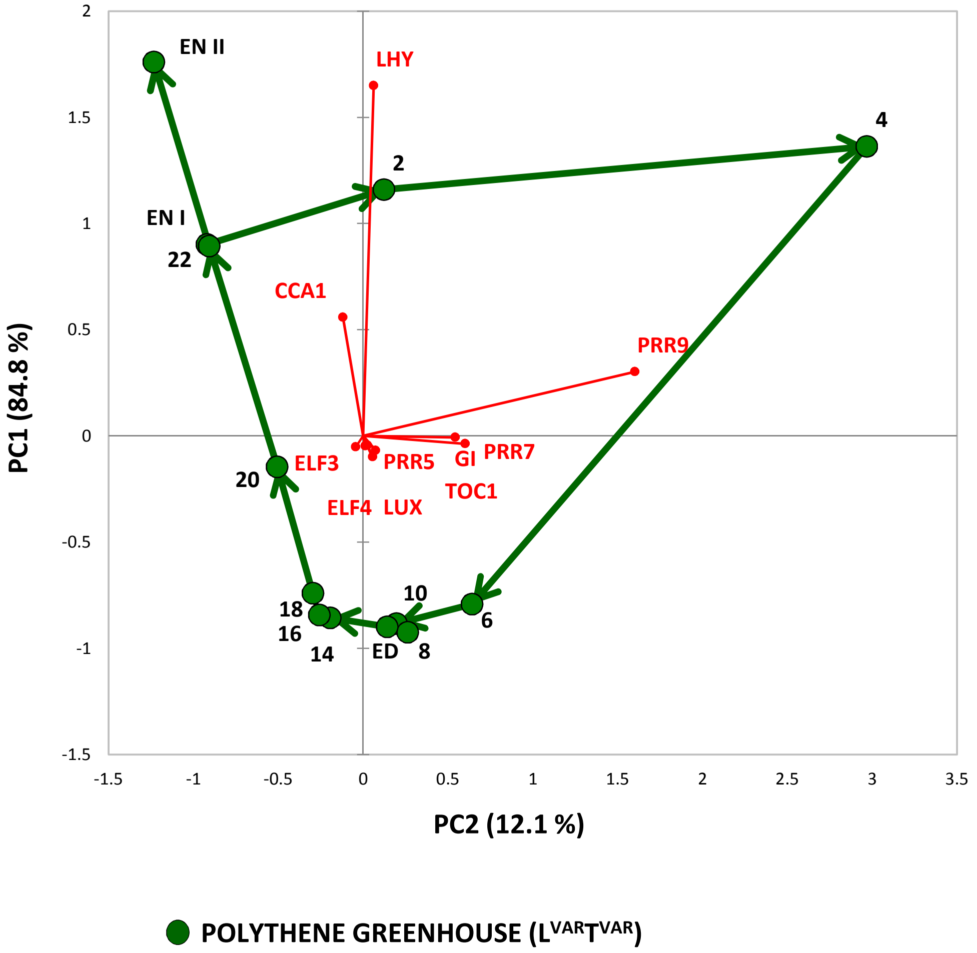

Supplemental Fig. S3. Principal component analysis (PCA) of core circadian clock gene data from variable light and variable temperature ($L^{VAR}T^{VAR}$) grown Arabidopsis plants (supporting information to Fig. 1). PCA of core clock genes from plants grown around the vernal equinox in 2015 in a naturally illuminated polythene greenhouse with variable temperature ($L^{VAR}T^{VAR}$, green circles). Numbers indicate the time of harvest in hours after dawn (zeitgeber time, ZT); ED, end of day (ZT12); EN I, end of preceding night (ZT0); EN II, end of night (ZT24), and the diurnal trajectory is indicated by the arrows. The percentages of total variance represented by principal component 1 (PC1) and principal component 2 (PC2) are shown in parentheses. The loadings of individual genes in PC1 and PC2 are shown in red.

(A) Sugar phosphates

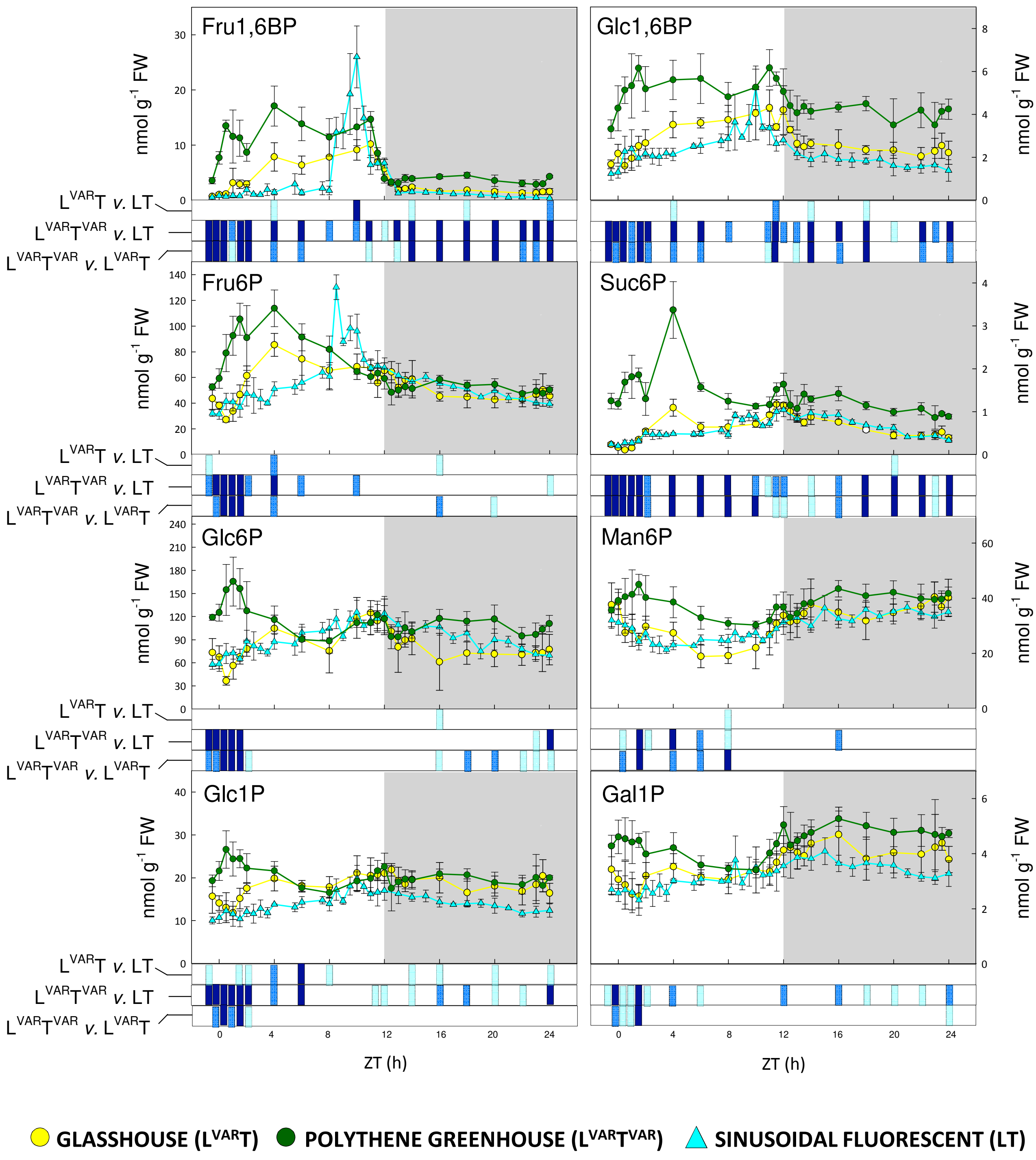

Supplemental Fig. S4. **Diel profiles of metabolites in different growth regimes** (supporting information to Fig. 2). *Arabidopsis thaliana* Col-0 plants were grown in a naturally illuminated and temperature controlled glasshouse ($L^{VAR}T$; yellow circles), a naturally illuminated polythene greenhouse with a less controlled temperature ($L^{VAR}T^{VAR}$; green circles) around the vernal equinox in 2015 and in a controlled environment chamber with a 12-h photoperiod and DLI of 12 mol m^{-2} d^{-1}. Artificial illumination was provided by white fluorescent tubes with a sinusoidal light profile (LT; cyan triangles). Rosettes were harvested from 4-week-old plants throughout a 24-h diurnal cycle for analysis of: (A) sugar phosphates; (B) TCA cycle intermediates; (C) major amino acids and Gln:Glu ratio; (D) aliphatic and aromatic minor amino acids; (E) other minor amino acids. Data are mean ± SD (n=4). At each time point, significant differences (by one-way ANOVA) between each pair of growth regimes are indicated by different colours: P<0.05=light blue, P<0.01=blue, P<0.001=dark blue.
ZT, zeitgeber time (hours after dawn). Panels (B), (C), (D) and (E) are on the following pages.

Supplemental Fig. S4 (continued). (B) TCA cycle intermediates

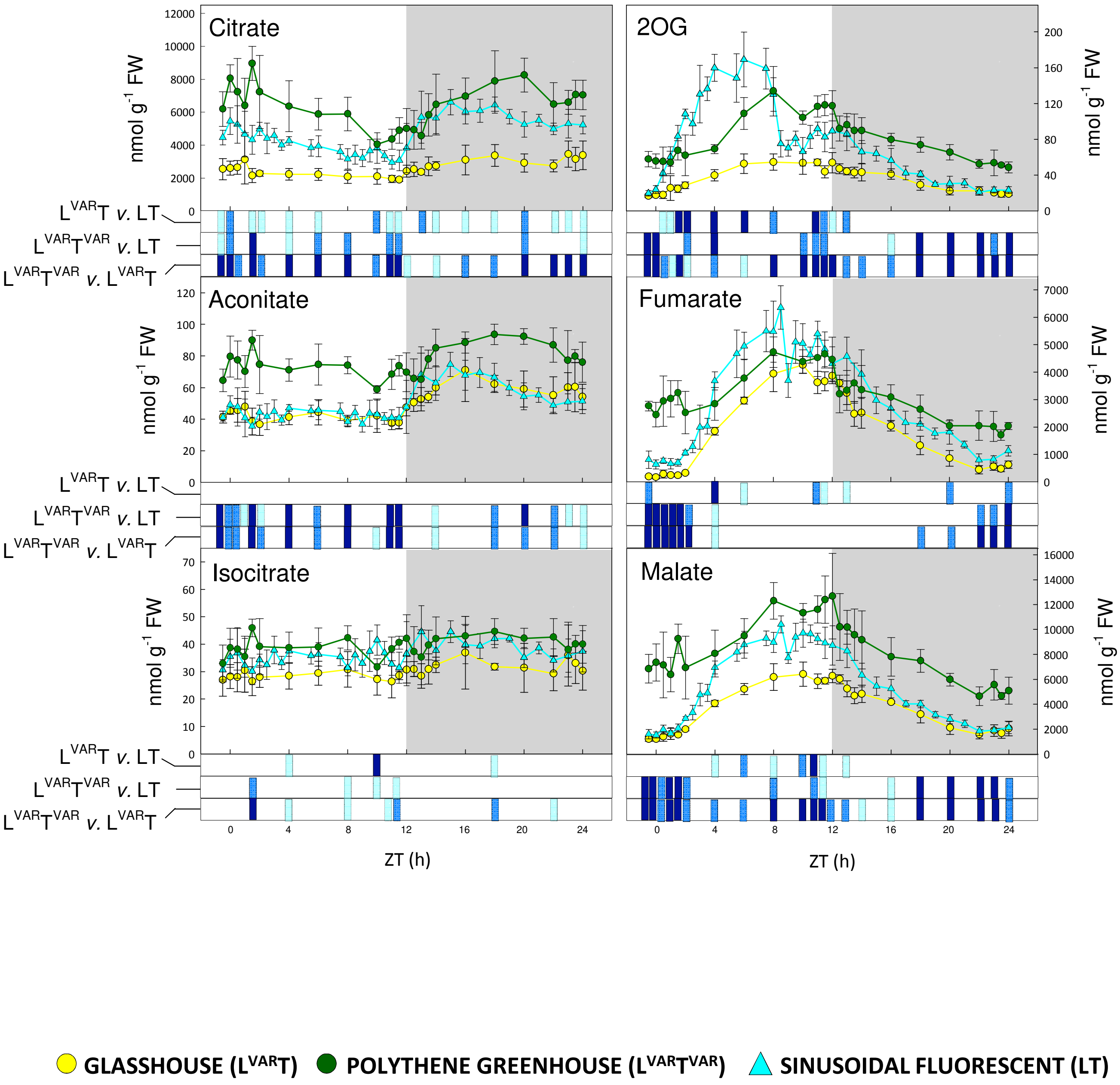

Supplemental Fig. S4 (continued). (C) Major amino acids and Gln:Glu ratio

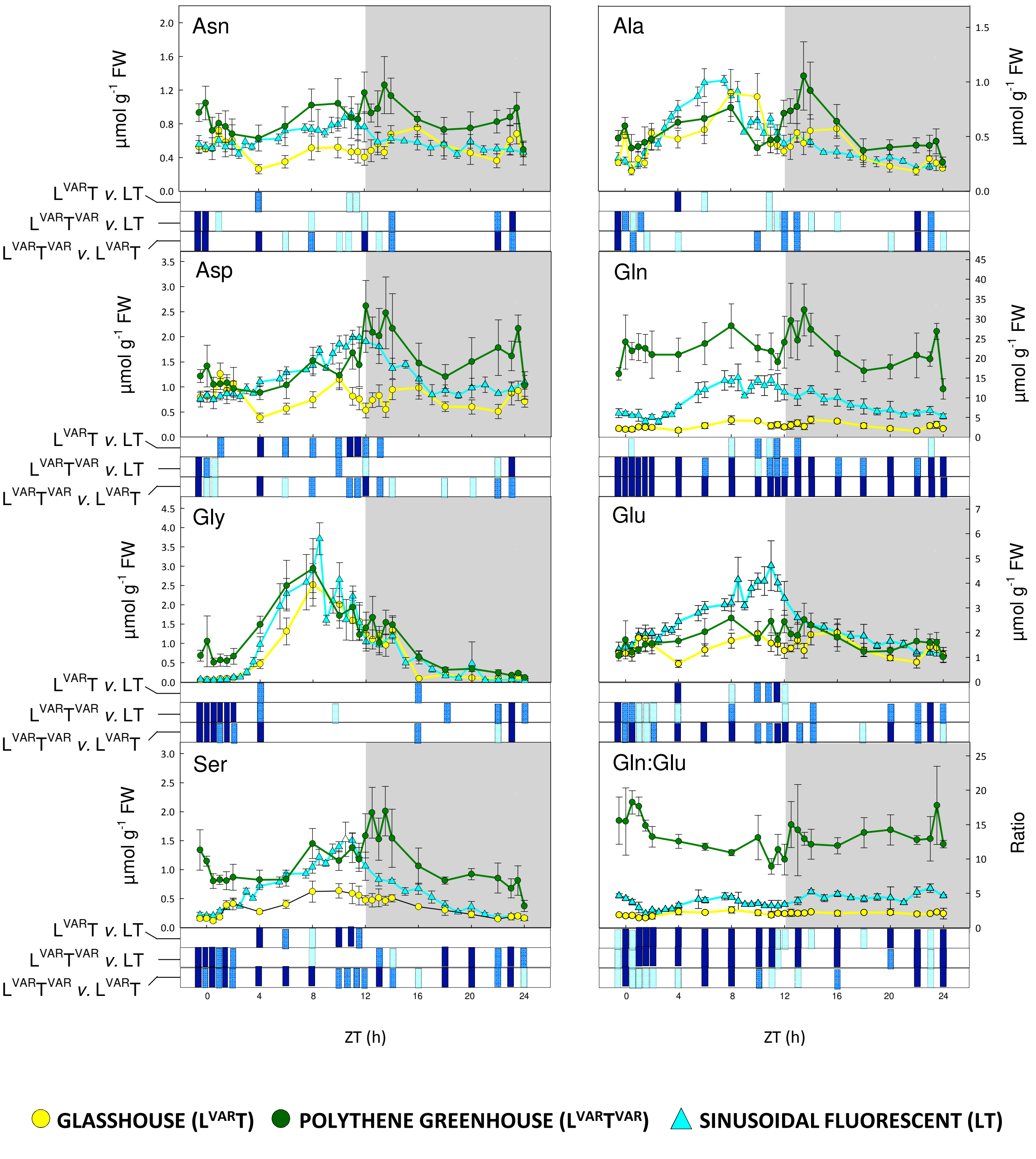

Supplemental Fig. S4 (continued). (D) Aliphatic and aromatic minor amino acids.

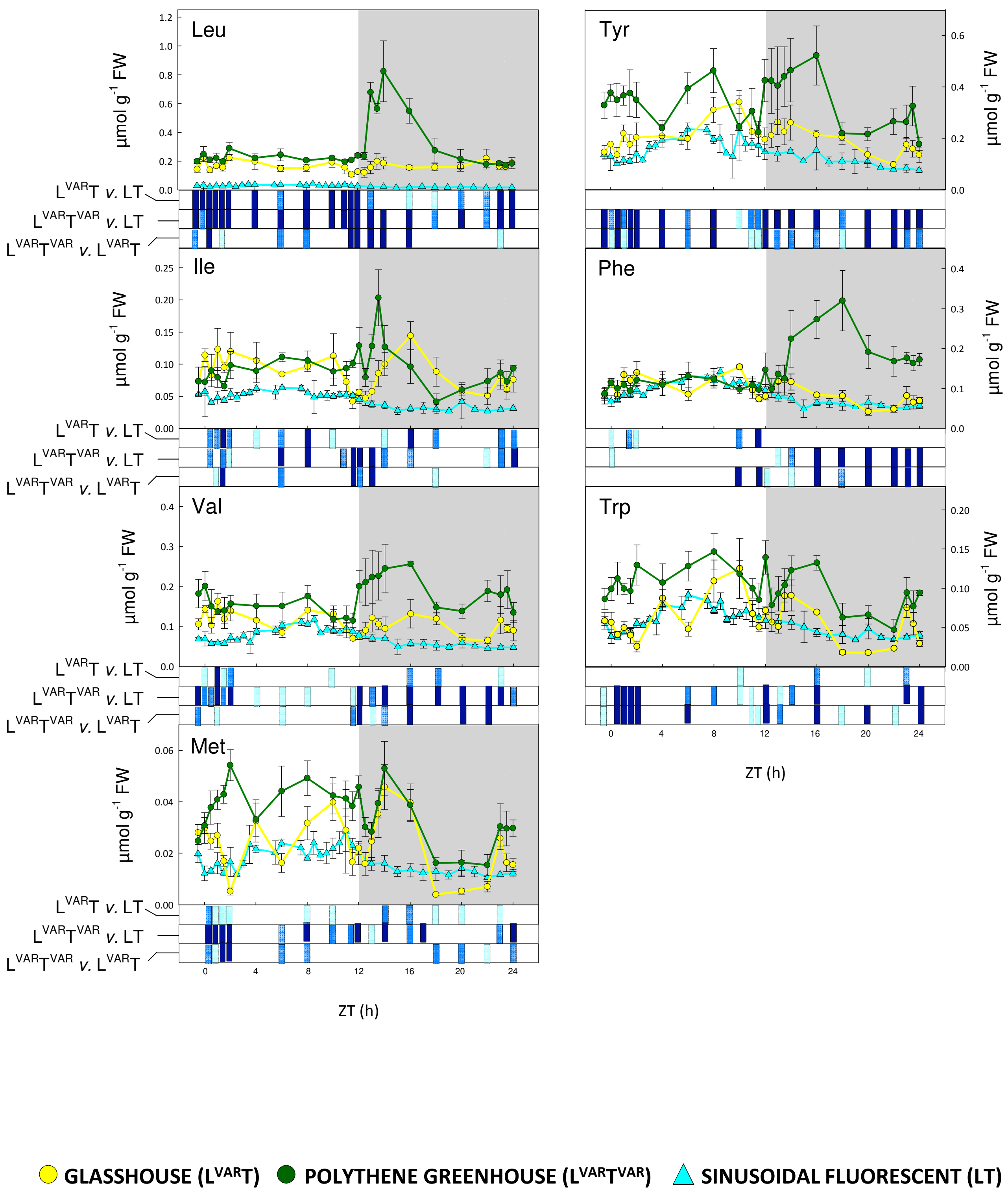

Supplemental Fig. S4 (continued). (E) Other minor amino acids. Orn, ornithine; Eta, ethanolamine.

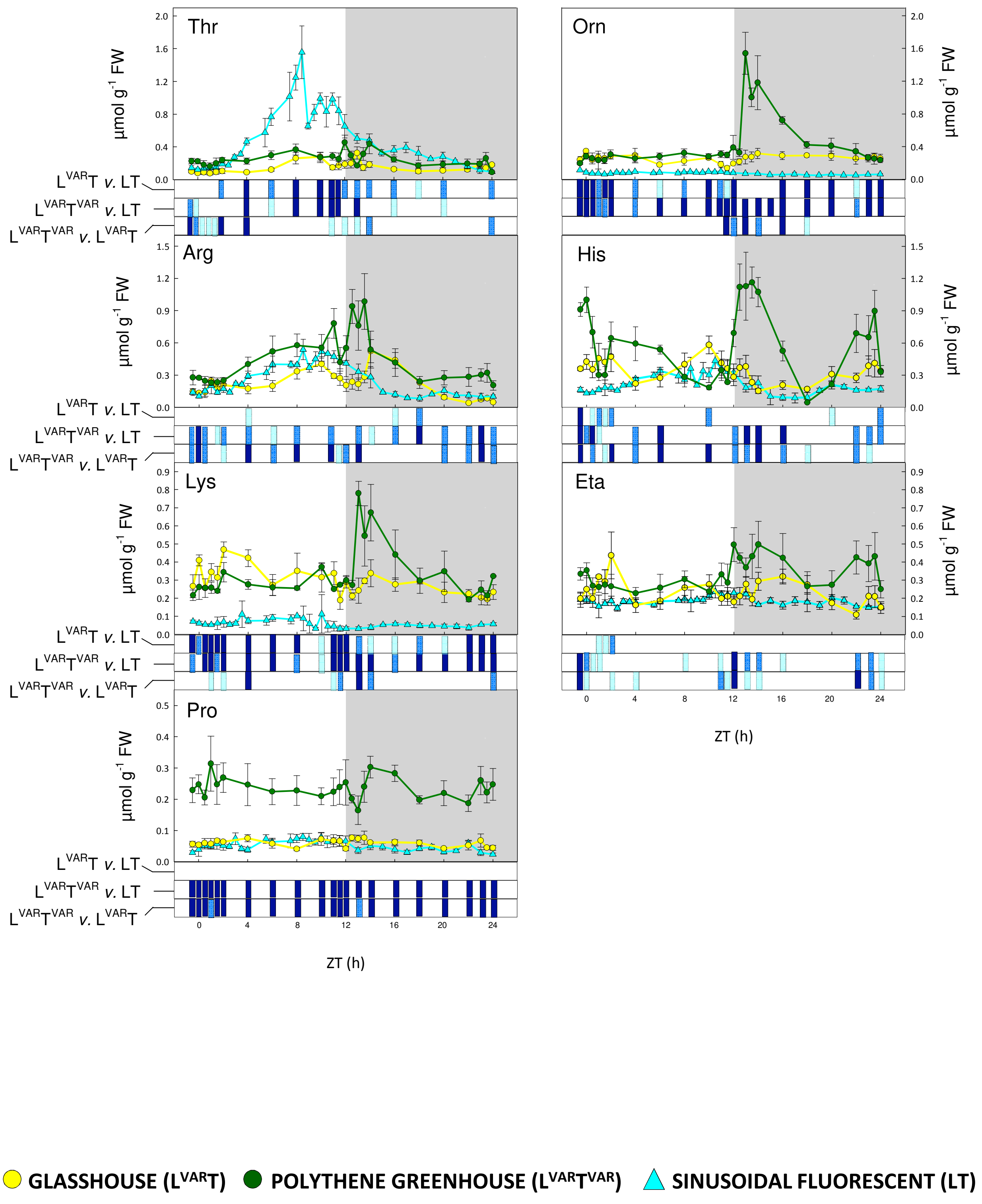

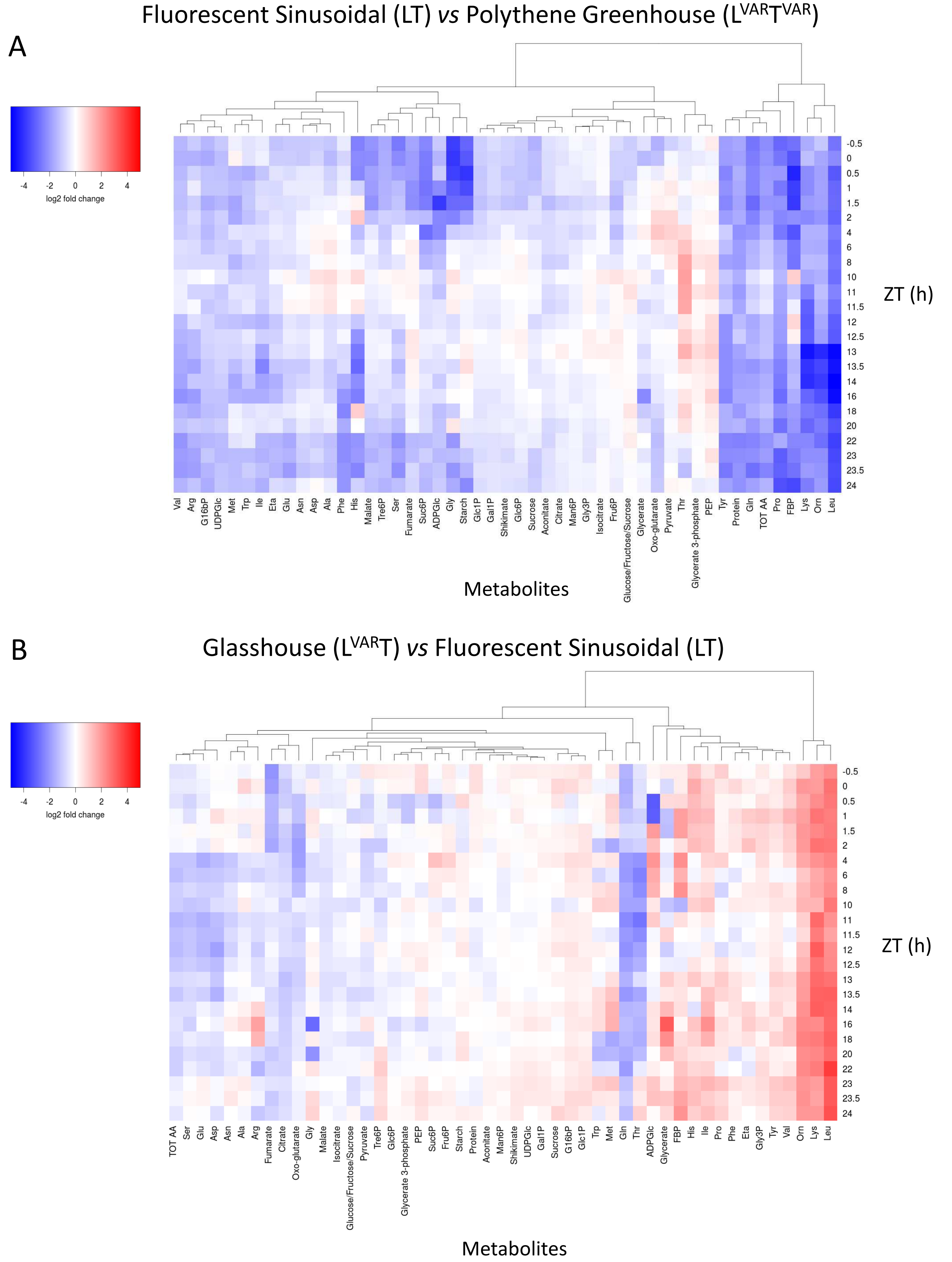

Supplemental Fig. S5. **Diel time series of metabolite levels compared between growth regimes:** (A) LT *versus* L^{VAR}T^{VAR} and (B) L^{VAR}T *versus* LT (supporting information to Fig. 3). Color key: low values – blue, high values – red. ZT, zeitgeber time (hours after dawn).

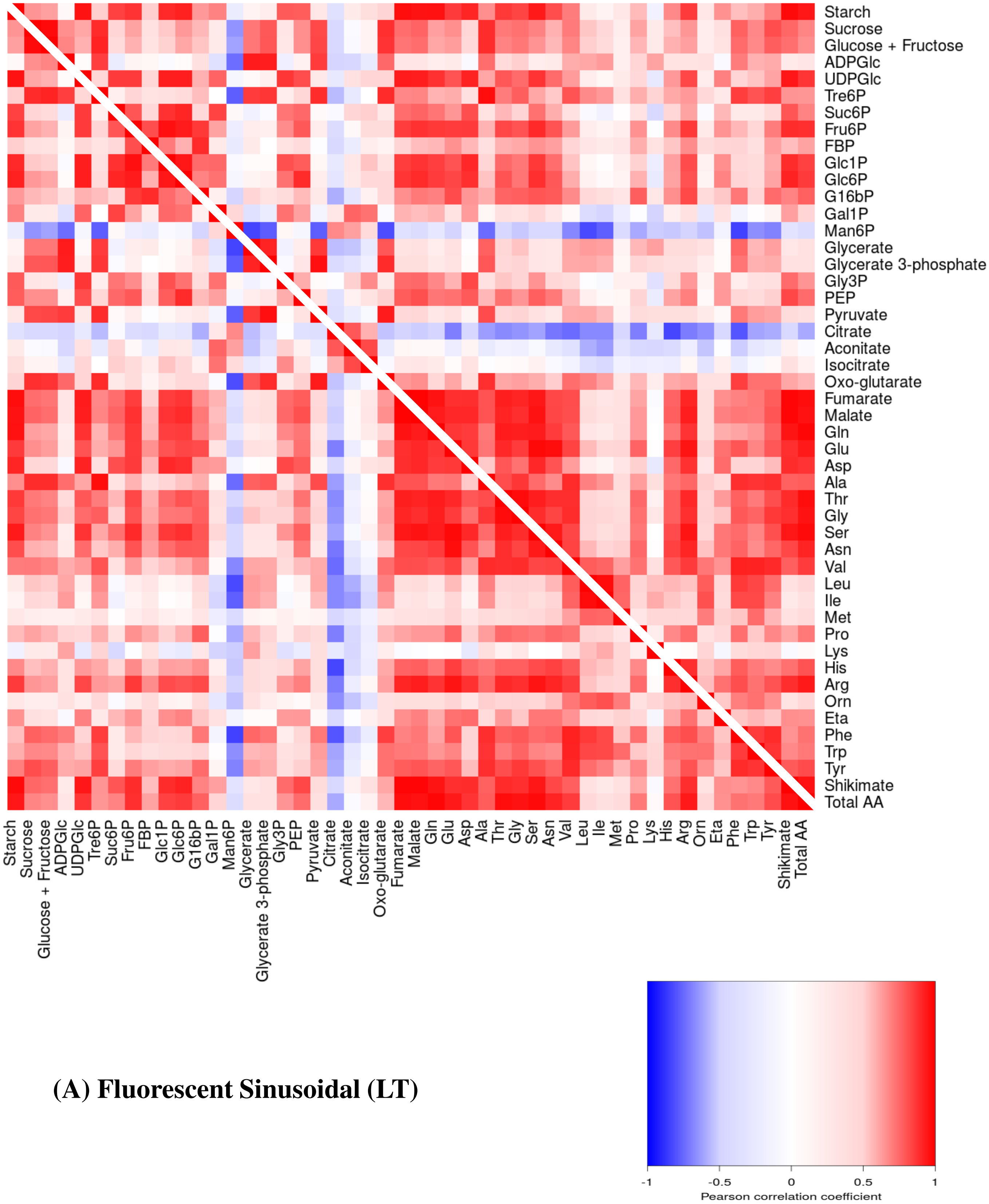

(A) Fluorescent Sinusoidal (LT)

Supplemental Fig. S6. **Diel time series of different metabolites compared within a given growth regime**: A) LT; (B) $L^{VAR}T$; (C) $L^{VAR}T^{VAR}$ (supporting information to Fig. 5). The results are presented as a heat map with the correlation score indicated by the shading: red, positive correlation; blue, negative correlation. The color scale is logarithmic and was selected such that the majority of non-significant correlations ($p < 0.05$) were not assigned any colour. Metabolites are grouped by category. Panels (B) and (C) are on the following pages.

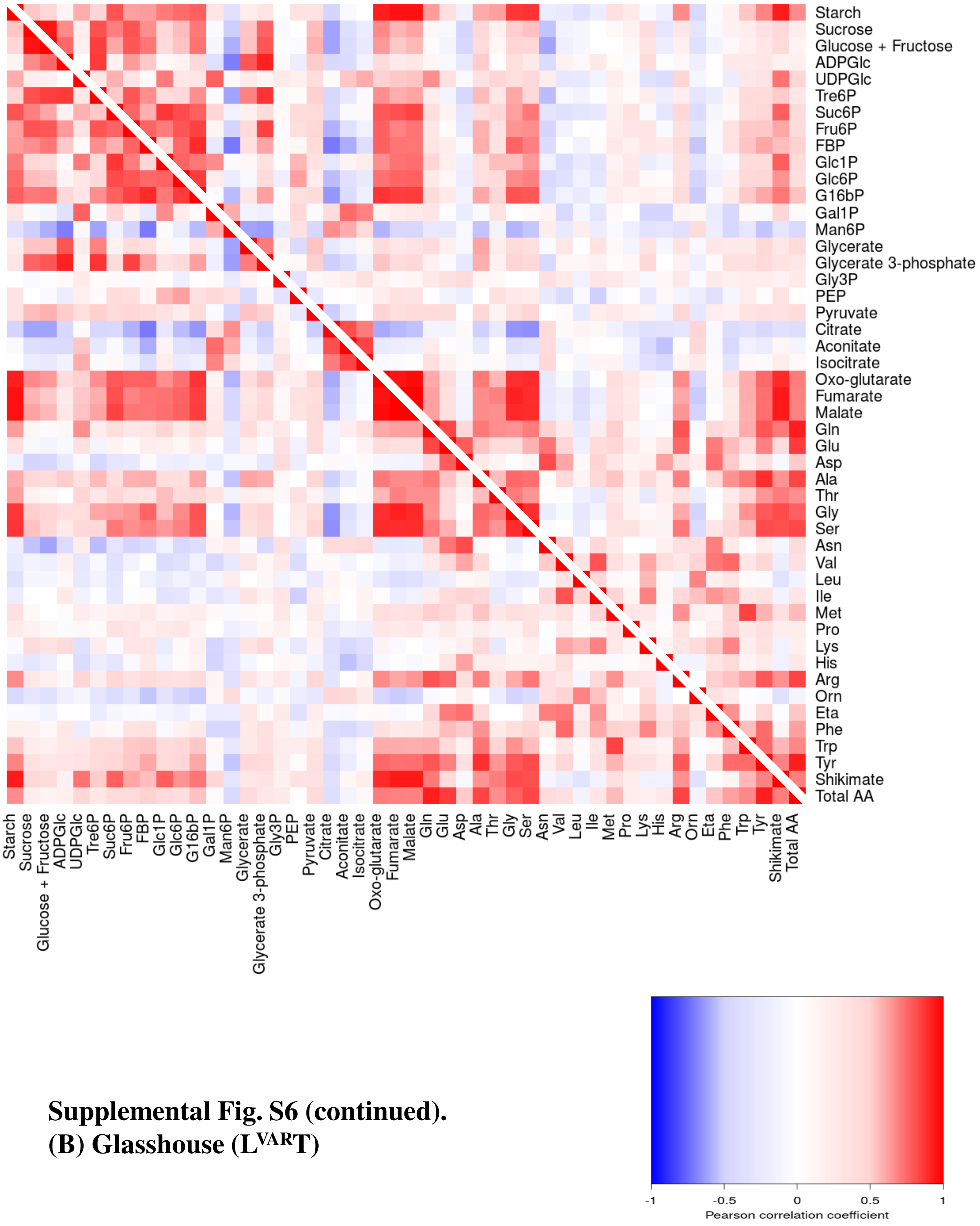

Supplemental Fig. S6 (continued).
(B) Glasshouse ($L^{VAR}T$)

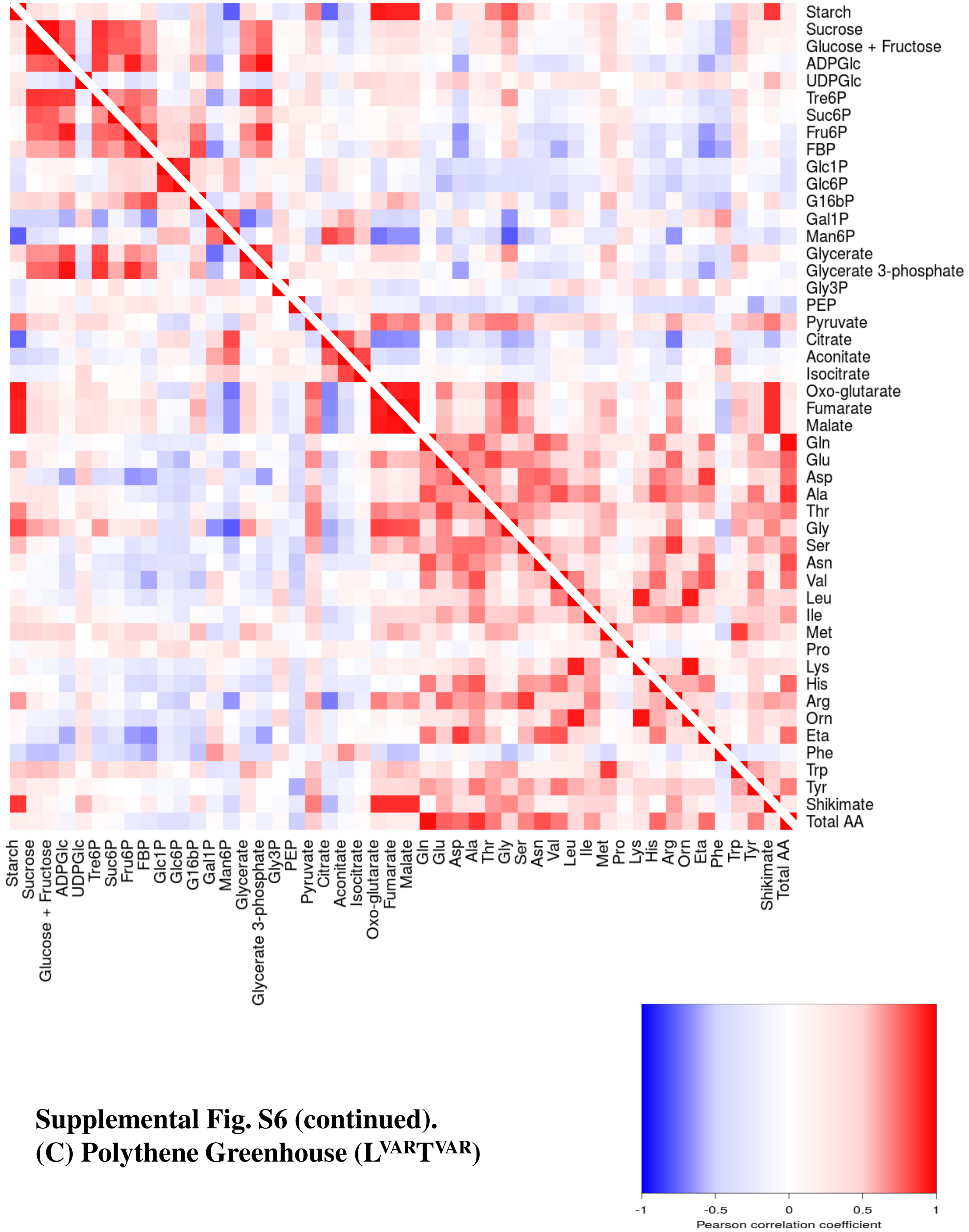

Supplemental Fig. S6 (continued).
(C) Polythene Greenhouse ($L^{VAR}T^{VAR}$)

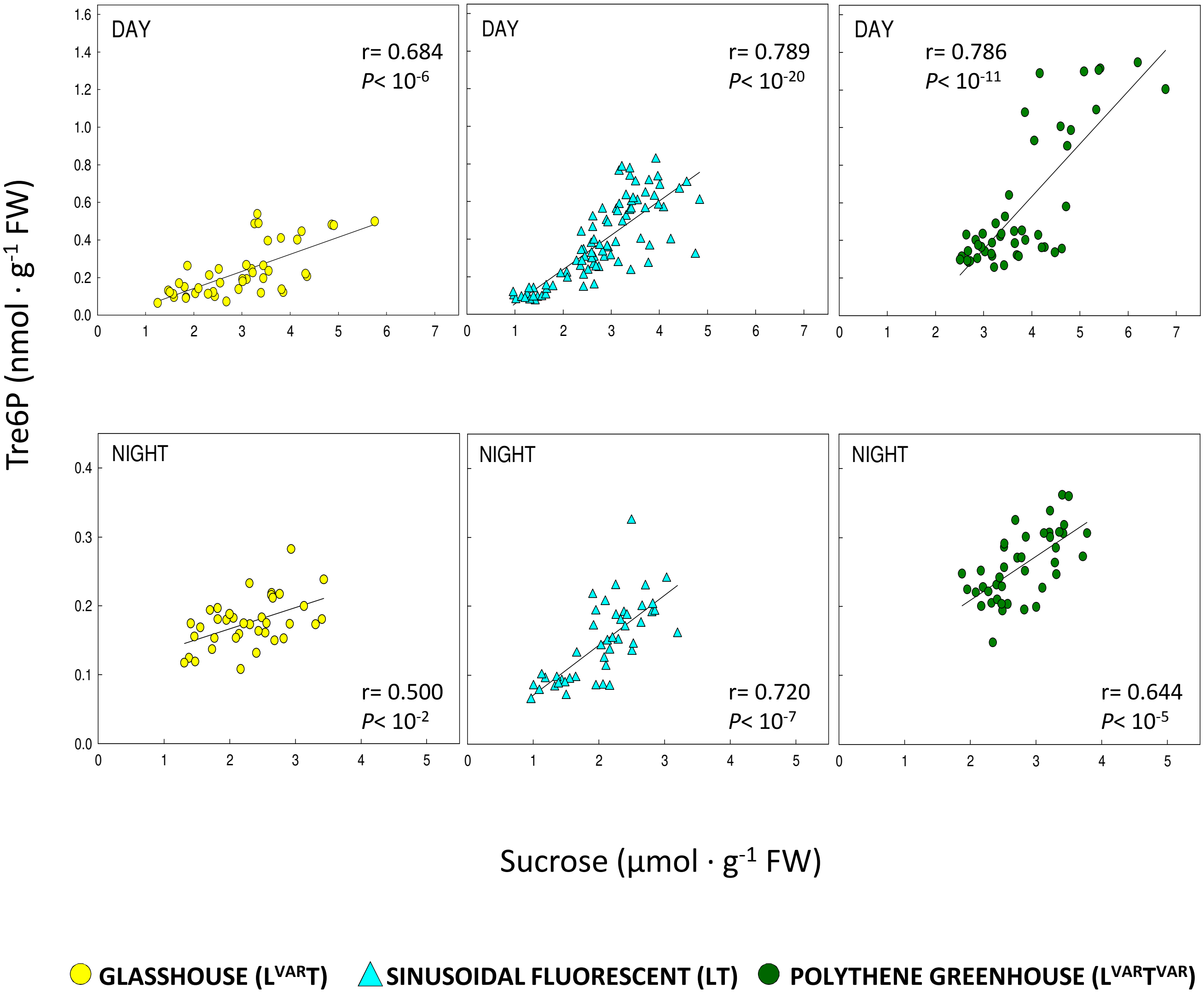

Supplemental Fig. S7. **Correlation between Tre6P and sucrose in different growth regimes**. The Pearson correlation coefficient (r) and the p-value (*P*) are shown.

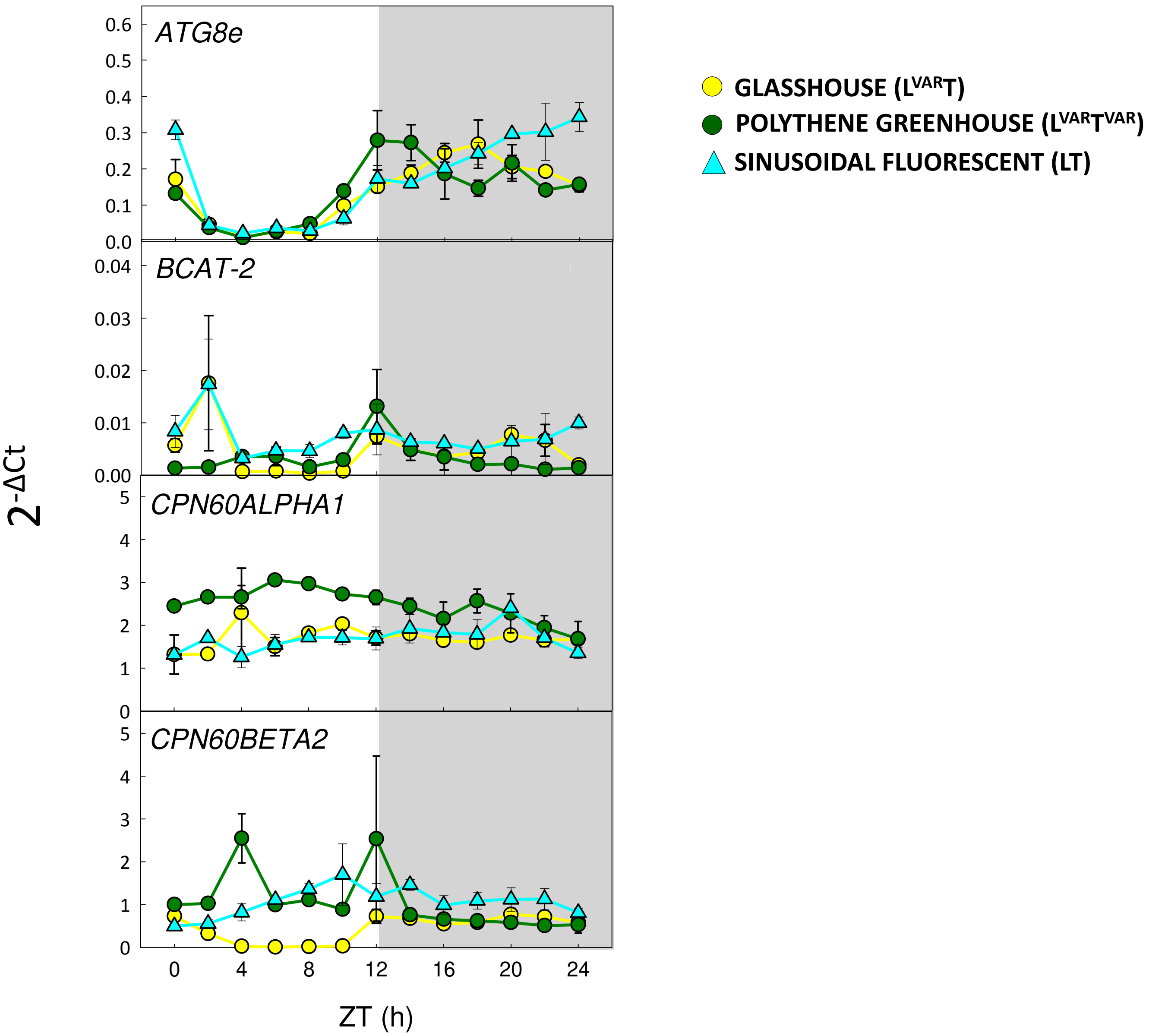

Supplemental Fig. S8. Transcript abundance for C-starvation marker genes. Transcript abundances were analysed by qRT-PCR in the same material as that used for metabolite analyses. Relative expression values were calculated as $2^{-\Delta Ct}$ for each sample where ΔCt indicates the difference from the Ct values of the different tested genes and the geometric mean of the Ct values of all reference genes. Data are mean ± SD (n=2). ZT, zeitgeber time (hours after dawn).

ATG8e, Autophagy-related protein 8e; *BCAT-2, Branched-chain-amino-acid aminotransferase 2*; *CPN60ALPHA1, Chaperonin-60alpha1; CPN60BETA2, Chaperonin-60beta2*.

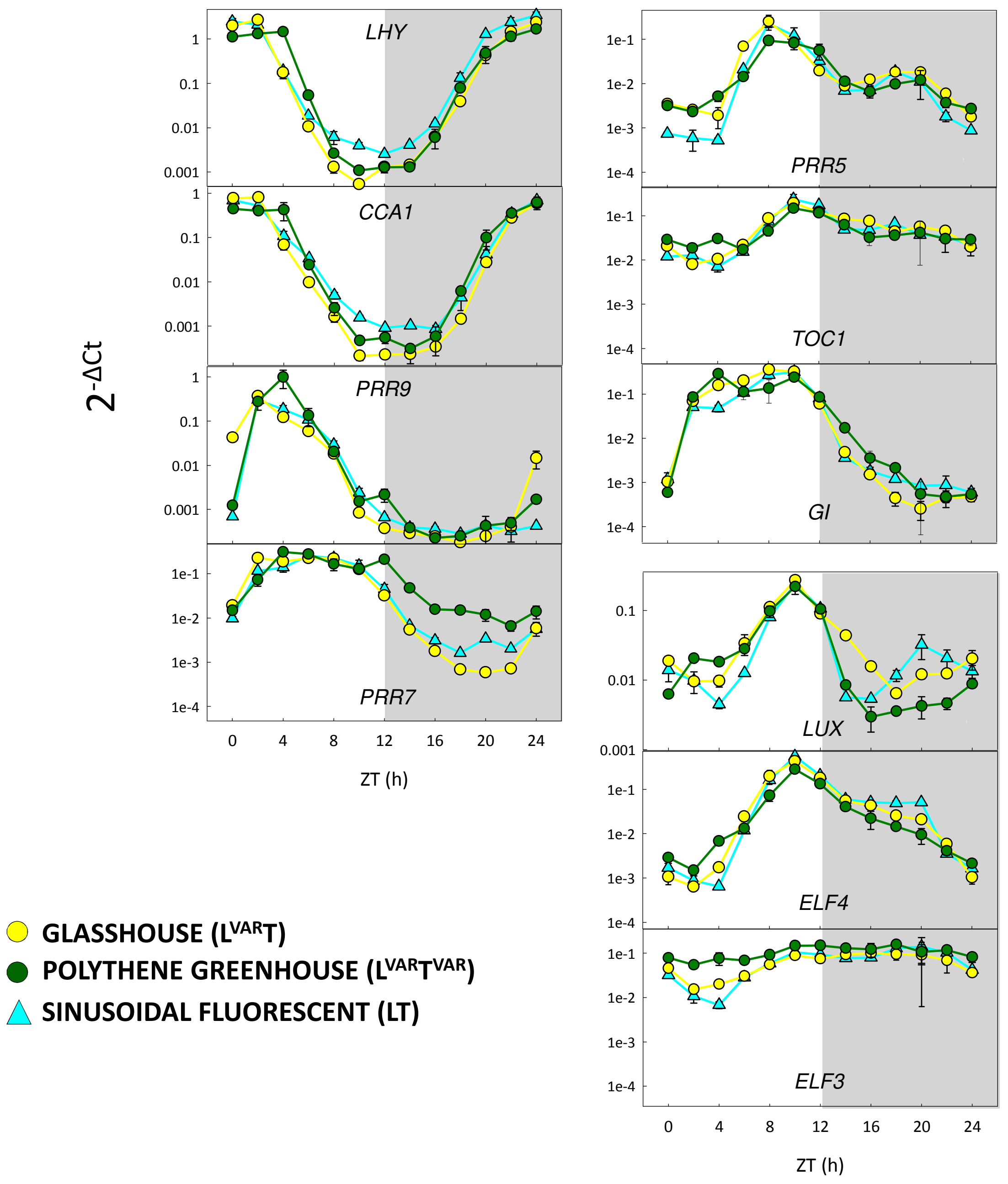

Supplemental Fig. S9. Log scale plots of diel transcript abundance of core circadian clock genes (supporting information to Fig. 6). Transcript abundance was analysed by qRT-PCR in the same material as that used for metabolite analyses**.** Relative expression values were calculated as $2^{-\Delta Ct}$ for each sample where ΔCt indicates the difference from the Ct values of the different tested genes and the geometric mean of the Ct values of all reference genes. Data are mean ± SD (n=2) and are shown on a log scale. ZT, zeitgeber time (hours after dawn).

LHY, LATE ELONGATED HYPOCOTYL; *CCA1, CIRCADIAN CLOCK ASSOCIATED1*; *PRR9, PRR7, PRR5*, *PSEUDO-RESPONSE REGULATOR9, 7* and *5*; *TOC1, TIMING OF CAB EXPRESSION1*; *GI, GIGANTEA; LUX, LUX ARRHYTHMO*; *ELF4, ELF3, EARLY FLOWERING4* and *3*.

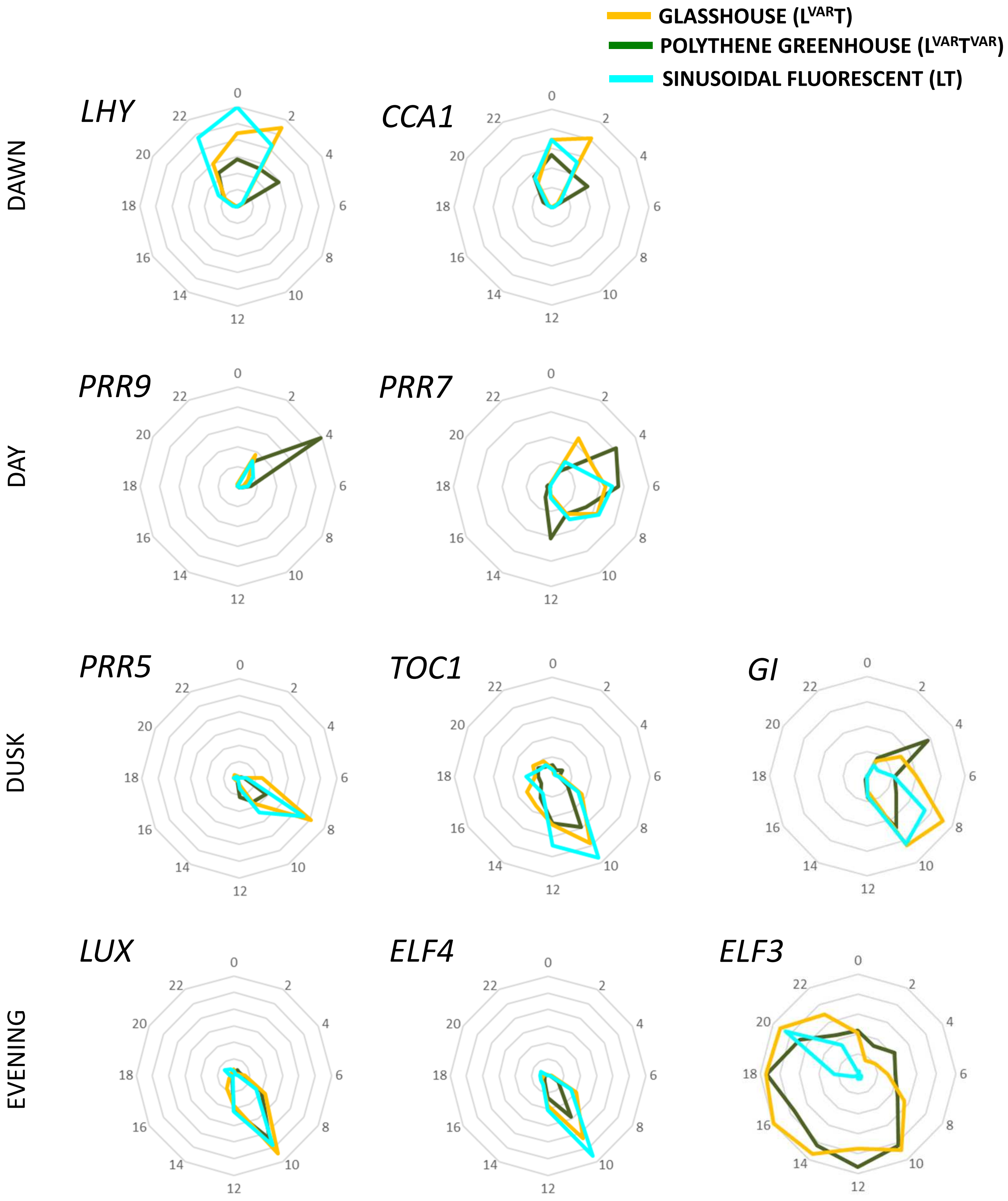

Supplemental Fig. S10. Radar plots of transcript abundance of core circadian clock genes (supporting information to Fig. 6). The data from Fig. 6 are replotted to display peak time. The scale is linear, and the outer boundary represents the highest expression level of a given gene in the three growth regimes. The time points are ordered in a clockwise manner around the plot, with ZT0 (dawn) at the top.

LHY, LATE ELONGATED HYPOCOTYL; CCA1, CIRCADIAN CLOCK ASSOCIATED1; PRR9, PRR7, PRR5, PSEUDO-RESPONSE REGULATOR9, 7 and *5; TOC1, TIMING OF CAB EXPRESSION1; GI, GIGANTEA; LUX, LUX ARRHYTHMO; ELF4, ELF3, EARLY FLOWERING4* and *3.*
